# Minimal conditions for solidification and thermal processing of colloidal gels

**DOI:** 10.1073/pnas.2215922120

**Published:** 2023-06-12

**Authors:** Scott M. Fenton, Poornima Padmanabhan, Brian K. Ryu, Tuan T. D. Nguyen, Roseanna N. Zia, Matthew E. Helgeson

**Affiliations:** ^a^Department of Chemical Engineering, University of California Santa Barbara, Santa Barbara, CA 93106; ^b^Department of Chemical Engineering, Rochester Institute of Technology, Rochester, NY 14623; ^c^Department of Chemical Engineering, Stanford University, Stanford, CA 94305

**Keywords:** colloids, gels, thermal processing

## Abstract

Colloidal gelation is used to form processable soft solids from a wide range of functional materials. Although multiple gelation routes are known to create gels of different types, the microscopic processes during gelation that differentiate them remain murky. A fundamental question is how the thermodynamic quench influences the microscopic driving forces of gelation, and determines the threshold or minimal conditions where gels form. We present a method that predicts these conditions on a colloidal phase diagram, and mechanistically connects the quench path of attractive and thermal forces to the emergence of gelled states. Our method employs systematically varied quenches of a colloidal fluid over a range of volume fractions to identify minimal conditions for gel solidification. The method is applied to experimental and simulated systems to test its generality toward attractions with varied shapes. Using structural and rheological characterization, we show that all gels incorporate elements of percolation, phase separation, and glassy arrest, where the quench path sets their interplay and determines the shape of the gelation boundary. We find that the slope of the gelation boundary corresponds to the dominant gelation mechanism, and its location approximately scales with the equilibrium fluid critical point. These results are insensitive to potential shape, suggesting that this interplay of mechanisms is applicable to a wide range of colloidal systems. By resolving regions of the phase diagram where this interplay evolves in time, we elucidate how programmed quenches to the gelled state could be used to effectively tailor gel structure and mechanics.

## Significance

Colloidal gels (solid particle networks formed by physical interparticle bonds) are found in materials spanning food and consumer products, coatings, and structural materials. Despite this ubiquity, we lack generalized theories to accurately predict the minimal conditions required for gelation. This is due both to the multiple mechanisms by which gels can form, and to the lack of unambiguous methods for determining where and when gels exist within a phase diagram. In this work, we develop an unambiguous method for determining the minimal conditions for gelation. The results unify different gelation mechanisms into a single framework, identify the importance of time in setting gelation behavior, and provide an outlook for using thermal processing to engineer the properties of colloidal solids.

Colloidal gels are soft, deformable materials comprising a solid particle network interpenetrated by solvent-filled pores. The network itself consists of microscopically small particles, droplets, bubbles, macromolecules, or proteins bonded together into strands of varying length and thickness, and forms when attractive forces between colloids trigger aggregation and assembly into a structure with persistent solid-like mechanics. Gelation is routinely used to form materials as diverse as food products (1, 2), industrial coatings, building materials (foams and concrete) (3–5), and biomedical products for drug delivery and tissue scaffolding (6).

An important effort for both the application and theoretical modeling of gels has been to understand how gel properties relate to their morphology and microstructure, resulting in a set of robust techniques for interrogating gel microstructure to predict their rheology, and vice versa (3, 6–17). A natural next step is to devise techniques that intervene in the gelation process to form certain gel structures (or discover new ones) with specific or exceptional mechanical properties. Predictive theory for the gel formation process itself, and how it sets gel properties, is thus an important goal of both fundamental and applied interest. However, existing equilibrium theories for colloidal phase transitions fail to predict gelation. The conventional approach, involving the placement of state points where gels form on a conventional phase diagram, can be fraught with uncertainty due to difficulties in identifying the density of the gelled “phase”. Once obtained, however, such state points offer opportunities to connect structure–property relationships with mechanistic understanding of gelation. Overall then, this approach aims to identify the locus of state points that minimally satisfies the conditions required to form a gel, i.e., the “gel line,” and to rationalize its location in the phase diagram through a particular mechanism(s) of gelation.

This approach has resulted in a large family of state diagrams, with gel lines located in different regions of the phase diagram relative to equilibrium phase boundaries for different materials (6). These many possibilities were previously rationalized by distinct, material-specific mechanisms of gelation, each with their own associated phenomenology. One such mechanism is percolation, which successfully describes chemical gelation (i.e., network formation by irreversible bonds) (18, 19), and more recently has been extended to describe physical gelation (network formation through reversible bonds) (20). Extensions of percolation theory to colloidal gelation have attempted to predict the occurrence of gelation from various microscopic definitions of percolation. One of these is connectedness percolation—in particular, isostatic percolation involving a continuous path of locally rigid clusters—with some studies reporting that the gel line compares well with the isostatic percolation threshold over a wide range of the colloidal phase diagram (9, 10, 21–23). Alternatively, Zhang et al. extended ideas of rigidity percolation—i.e., the ability of a percolating structure to globally transmit stress—to simulations of colloidal gels, and proposed that so-called correlated rigidity percolation, whereby a majority of fluctuating states possess a rigid percolating structure, is more closely associated with gelation (24).

A second proposed mechanism is arrested phase separation, in which thermodynamic instability of a homogeneous colloidal fluid produces bicontinuous networks of thick, colloid-rich strands coexisting with colloid-dilute pores (7). Support for this mechanism has involved comparing the location of the gel line with the underlying equilibrium fluid–fluid (or fluid-solid) phase boundary. Early work showed that the experimentally observed gel line for colloids with attractions mediated by depletion of nonadsorbing polymers followed closely to the theoretically predicted equilibrium vapor-liquid phase boundary for colloid densities near the critical point (12, 25). However, this coincidence of gelation and phase separation is not necessarily evident in other systems with apparently similar-shaped interaction potentials. For example, in globular protein systems gelling through short-ranged attractions, it was determined that the gel line lies at quenches significantly below the equilibrium phase boundary (26, 27). It is thus unclear whether phase separation is necessary or sufficient to form colloidal gels in a particular system.

A third proposed mechanism is the glass transition, in which dramatic slowing of dynamics produces a persistent, nonequilibrium solid-like state. This transition can be located on the equilibrium phase diagram as a glass line bounding the conditions where dynamic arrest occurs, usually defined by the divergence of viscosity or structural relaxation time (6, 28). Combinations of mode-coupling theory (MCT) and Brownian dynamics simulations for short-ranged attractive colloids established that the attractive glass line—where glassy arrest occurs through interparticle bonding—crosses the dense side of the equilibrium fluid–fluid spinodal boundary at a temperature, *T*_*g*_^*sp*^, below the critical point (6, 28–32). This suggests that phase separation is not sufficient to form a gel. For quenches into the region of phase instability, quenches above *T*_*g*_^*sp*^ produced uninterrupted phase separation, whereas quenches below *T*_*g*_^*sp*^ exhibited glassy arrest of the dense phase (6, 26, 27, 30, 33). It was therefore proposed that gelation of a homogeneous fluid at high densities occurs due to formation of an attractive glass, and that *T*_*g*_^*sp*^ sets the minimal conditions for gelation under quenches into the region of phase instability. However, the shape and exact location of the gel line within the phase coexistence region were not resolved. Moreover, subsequent studies (7, 34, 35) showed that, unlike colloidal glasses formed in the homogeneous fluid phase, gels once formed within the region of phase instability continue to coarsen over time, suggesting that gelation cannot be fully explained by the intersection of the glass transition line with the phase boundary. It is therefore clear that the glass transition alone is insufficient to predict the formation of colloidal gels in regions of colloidal phase instability.

The inadequacy of any one mechanism to predict gelation across a range of material systems has motivated more recent attempts to unify the diverse range of observed behavior, to varying degrees of success (10, 26, 30, 32, 36–38). In experiments, Harich et al. (33) proposed that gelation in a colloidal system with depletion-induced attractions was set by an interplay of percolation, phase separation, and the glass transition. A similar situation was proposed by Sedgwick et al. (39) for a globular protein system. However, inconsistencies in both studies between experimentally and theoretically obtained state diagrams necessitate finer scrutiny on the potential interplay of these mechanisms and how they set gelation, as well as techniques to better locate the gelation transition on experimental and simulated state diagrams.

More recent theoretical work has also aimed to address the inadequacy of any one specific mechanism to predict gelation. For example, cluster-based analogues of MCT aimed to explain gelation at more dilute colloidal volume fractions, *ϕ* (13, 40). Alternatively, it was proposed that the Noro–Frenkel extended law of corresponding states (ELCS) (41), which collapses the equilibrium phase behavior of colloidal systems with sufficiently short-ranged attractions using the reduced second virial coefficient, *B*_2_^*^, may similarly collapse the gel lines observed across different colloidal systems. Although this hypothesis appears to describe the gel lines observed in depletion-mediated gels with varying attraction range (12), it fails for other systems such as globular proteins (26).

The failure to develop a theory that unifies the mechanisms of gelation across different systems exposes a key limitation of the conventional approach—while individual mechanisms of gelation can be descriptive for materials whose gelation is established to be dominated by that mechanism, there is limited existing framework to predict where the gel line falls within the potentially large region of the phase diagram (involving quenches into the region of phase instability) where two or more of these mechanisms would be expected to predict gelation. The practical challenge of determining the gel point (the location of the gel line for a particular thermodynamic state) in experiment or simulation exposes a second limitation, in that the conventional approach is naïve to the influence of time on the gelation process. This forces arbitrary decisions regarding how long one must wait for a gel to form. To illustrate, we consider what happens as one performs successive gelation attempts involving quenches closer and closer to the gel point. Because gelation is a kinetic process (i.e., elasticity emerges over time), and the driving force for gelation is expected to increase with the depth of the thermal quench (i.e., the strength of a “bond”), we recognize that a system under such an asymptotic quench will require arbitrarily large amounts of time to solidify. Therefore, the “true” gel point is inaccessible to experimental or computational measurements, which must be performed over a finite time window. In systems where attractions are modulated by mixing of components (polymers, salts, etc.), this challenge is difficult to resolve due to the uncontrolled kinetics of quenching upon mixing (9).

For colloidal systems where attractions are thermoresponsive—including protein solutions (27, 42, 43), microgel suspensions (44–46), and polymer-colloid mixtures (11, 15, 16, 21, 22, 47–51)—controlled quenching is more feasible and can be used to more clearly identify gelled states within a colloidal phase diagram. Moreover, new approaches to interrogate and rationalize time-dependent gelation behavior offer the opportunity to use controlled thermal quenching as a means to intervene in the gelation process as it evolves over time. This could lead to strategies for sculpting gel structure and properties akin to the use of thermal processing to obtain rare or exceptional properties in multiphase metal alloys (52), ceramics (53), or polymers (54). For example, Cates and coworkers (13) posited that quenches at different rates could be used as a means of controlling gelation behavior when the time scales of phase separation intersect with the time scales of the quench.

Testing this hypothesis requires—at minimum—detailed knowledge of how phase separation, percolation, and glassy arrest compete with time and quench as one moves across the phase diagram. A critical starting point in this effort is to understand the location and nature of the gel line in thermoresponsive systems. Previous attempts relied on linear viscoelastic measurements during continuous temperature ramps (14), or after rapid temperature jumps followed by a fixed, predetermined aging protocol (21, 35, 49, 55). Often, these measurements are made under small amplitude oscillatory shear (SAOS), where one must choose whether to probe the material in time at fixed oscillation frequency, or with quasi-static frequency sweeps, where gelation is typically determined using the commonly employed Winter and Chambon criterion (56). Scattering measurements can also probe gelation by observing the temperature at which nonergodic states emerge (15, 35, 47). Many of these methods capture the mechanics of the system at an arbitrary time point (or range of time). However, because gelation is a kinetic process, such measurements can incorrectly predict the final state and mechanics of the system. Moreover, temperature ramp measurements result in predictions of the gel line that depend on the rate of quenching (14). Despite the number of techniques available to approximate the location of the gel line, there is no clear consensus for how one maps these techniques for an asymptotic approach (in time) to the gelled state onto an asymptotic quench (in thermodynamic state) to the gel line.

This ambiguity motivated us to develop a method for systematically locating the gel line that could be applied in combination with highly tunable model systems to confirm and extend previously proposed mechanisms of gelation from other model systems. The method uses rheological measurements to monitor solidification during controlled differential (temperature) quenches in colloid attraction strength, and asymptotic analysis to extrapolate conditions where a gel takes infinite time to form. We demonstrate our method on a previously established experimental system of nanoemulsions with thermoreversible colloidal attractions (11, 14, 16, 35, 50, 55, 57, 58) and in dynamic particle simulations involving variations in the interparticle potential. When combined with detailed structural and mechanical measurements, the results elucidate that the minimal gel line is set by a complex interplay of percolation, phase separation, and glassy arrest, which dissects the phase diagram into a number of regions with differing time-dependent gelation behavior that is conserved across interaction potentials of differing range. Determining the regions of the phase diagram in which phase separation is operative and sensitive to quench during gelation identifies general guidelines for the design of time-dependent quenches that achieve potentially large variations in ultimate gel structure and mechanics, paving the way for thermal processing of colloidal gels.

## Model Systems

### Experimental model system.

**Fig. 1.**
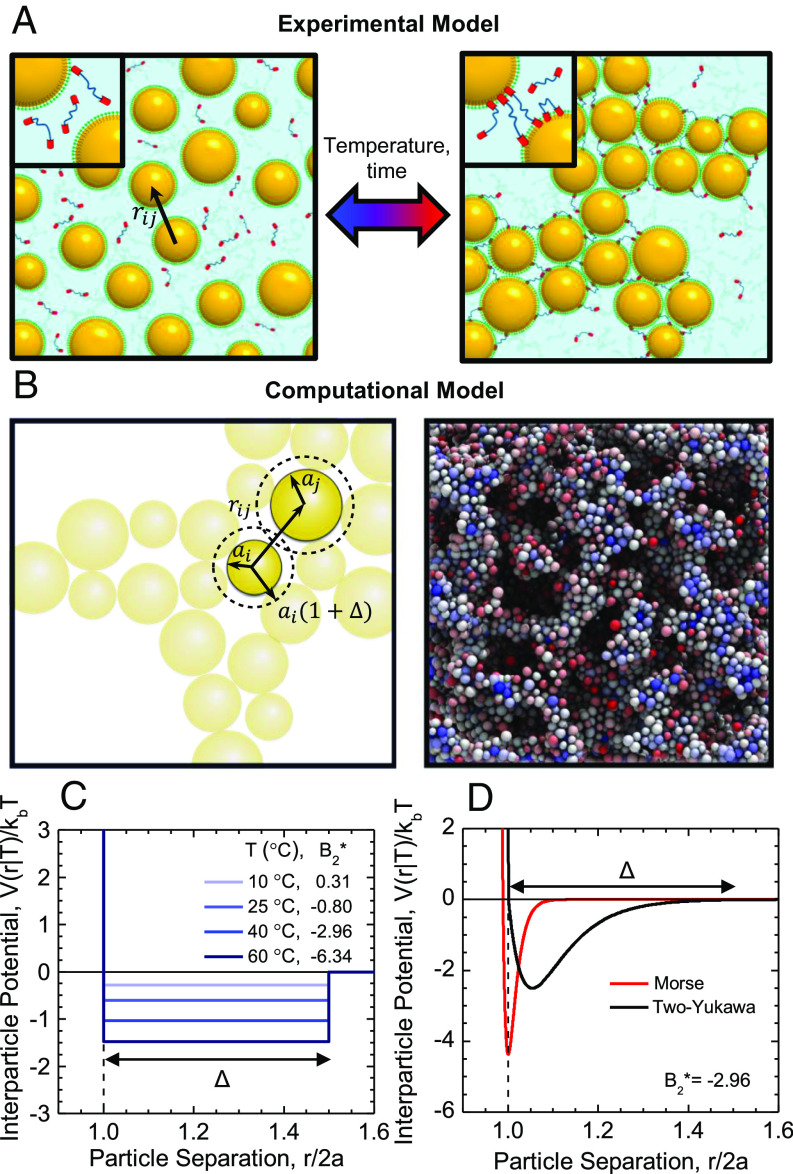
(*A*) Experimental nanoemulsion system composed of PDMS droplets dispersed in water with thermoresponsive PEGDA bridging polymers. Increasing temperature triggers adsorption of the hydrophobic linker end groups, resulting in interdroplet attractions and formation of an elastic network. (*B*) Computational model system composed of a polydisperse suspension of Brownian spheres with any two particles of size $a_{i}$ and $a_{j}$ separated by a distance $r_{\mathrm{ij}}$ that interact via an attractive interaction potential over a total interaction distance Δ. (*C*) The experimental droplet interactions can be modeled using a temperature-dependent square-well (T-SW) interaction potential with well width, *Δ*. The interaction potential shown here was extracted from SANS experiments on a dilute suspension of nanoemulsion droplets by Helgeson et al. (11). (*D*) The computational colloidal interactions are modeled using two different potential forms: a short-ranged Morse potential and a longer-ranged 2Y potential (compared here to the T-SW potential at an equivalent *B*_2_^*^ of −2.96) to determine whether the qualitative features of the gel line depend on the shape and range of the interaction potential and to corroborate experimental results.

We choose as a model thermoresponsive colloidal system the oil-in-water nanoemulsion system developed by Helgeson et al. (11), in which interdroplet interactions arise from associative polymer bridging due to temperature-sensitive poly(ethylene glycol) diacrylate (PEGDA) linkers, resulting in effective interdroplet attractions that depend on the laboratory temperature (T) of the system (Fig. 1*A*). Because the interactions are temperature-dependent, they can be tuned rapidly using conventional external temperature control. Nanoemulsions can be formulated for a range of *ϕ* where the small droplet dimensions minimize light scattering, permitting access to microscopy and scattering measurements (35, 59).

To develop a corresponding interaction potential model, *V*(*r*|*T*), that describes the temperature-dependent droplet interactions as a function of interparticle separation, *r*, small angle neutron scattering (SANS) experiments were conducted previously on a dilute nanoemulsion sample over a range of temperatures (11). A square-well (SW) potential with fixed well width, *Δ*, set by the extended end-to-end distance of the polymer linkers was fit to the scattering intensity, I(q), with wavevector q, to determine the well depth, *V*_0_/*k*_*b*_*T*, of the potential for each lab temperature tested where *k*_*b*_ is Boltzmann’s constant (*SI Appendix* for details). The resulting temperature-dependent SW potential (T-SW) is shown in Fig. 1*C*. As temperature is increased, *V*_0_ increases monotonically with a sigmoidal temperature dependence (35). This allows conversion from laboratory temperature to $B_{2}^{*}$ using:

[1] $$\begin{array}{c} B_{2}^{*}\left(T\right)=\frac{B_{2}\left(T\right)}{B_{2,HS}}=-\frac{3}{\sigma^{3}}\int_{0}^{\infty }\left[e^{-V\left(r|T\right)/k_{b}T}-1\right]r^{2}dr, \end{array}$$

where *σ* is the particle diameter. By converting from temperature to $B_{2}^{*}$, direct quantitative comparisons between the experimental system and the simulation systems can be made.

### Computational model system.

We constructed an analogous computational model using the LAMMPS molecular dynamics package (*SI Appendix* for details), which consists of a suspension of nearly hard sphere colloids with average radius *a* in the freely draining limit (Fig. 1*B*). To mitigate challenges in modeling reversible colloidal gels, which possess a hierarchy of length scales, we construct the computational model with 750,000 particles in a periodically replicated cell that permits sampling of many length scales and time scales.

Two models for interparticle interactions were chosen, the Two-Yukawa (2Y) and Morse potentials (shown in Fig. 1*D*) (*SI Appendix* for functional forms and parameter values). The 2Y potential is commonly used to describe the combination of attractive and repulsive interactions that arise in charged colloidal particles solutions like micellar and globular protein solutions (26, 60, 61), as well as between colloids in polymer-colloid mixtures (62). The 2Y potential can also capture the qualitative features of the interactions present in the experimental nanoemulsion system, i.e., a hard sphere repulsion followed by an attraction arising from polymer bridging. The Morse potential was also implemented to determine whether the detailed shape and range of the interaction potential changes the qualitative features of the gelation transition. The Morse potential was also chosen because it has been well studied in previous molecular dynamics simulations on colloidal gelation (7, 34, 63, 64) to describe systems with depletion attractions.

The simulation system is ideal for corroborating experiments because particle interactions can be controlled precisely. It is also ideal because as we will show, it can be used to determine not only the gelation threshold but also the percolation threshold and the equilibrium phase boundary. Most previous studies have relied on different methods (a combination of theory, experiments, and simulation) to develop these lines. For instance, it is common to develop a gelation line from experiments using rheological measurements, but use theory with the proposed interaction potential to develop the phase boundary. A challenge with such an approach is that the comparison between the resulting gelation line and phase boundary depends on the chosen form of the interaction potential, resulting in ambiguity about the location of the gelation line relative to the equilibrium phase boundary (12, 33, 58).

## Results and Discussion

### Identifying the Gel Line.

There is currently no generally accepted method to precisely determine the gelation threshold for quench-controlled systems. As mentioned previously, past attempts involved an arbitrary observation time to test for gelation at a particular thermodynamic state. Because gelation takes time to occur, this imprecisely predicts the location of the gelation threshold, motivating the need for a method that is insensitive to the time over which the characterization is performed. Our method assumes that there exists a minimum quench depth above which a system will never gel, and this defines the minimal gel point for the material at a given volume fraction *ϕ*. In other words, we define the gel point (or the gel line, if conducted at several volume fractions) as the minimum quench required to observe a dominant elastic response given *infinite* observation time. Of course, it is infeasible in experiments and simulation to wait infinite time to characterize the state of the system, so we instead estimate the gelation threshold using an asymptotic extrapolation.

**Fig. 2.**
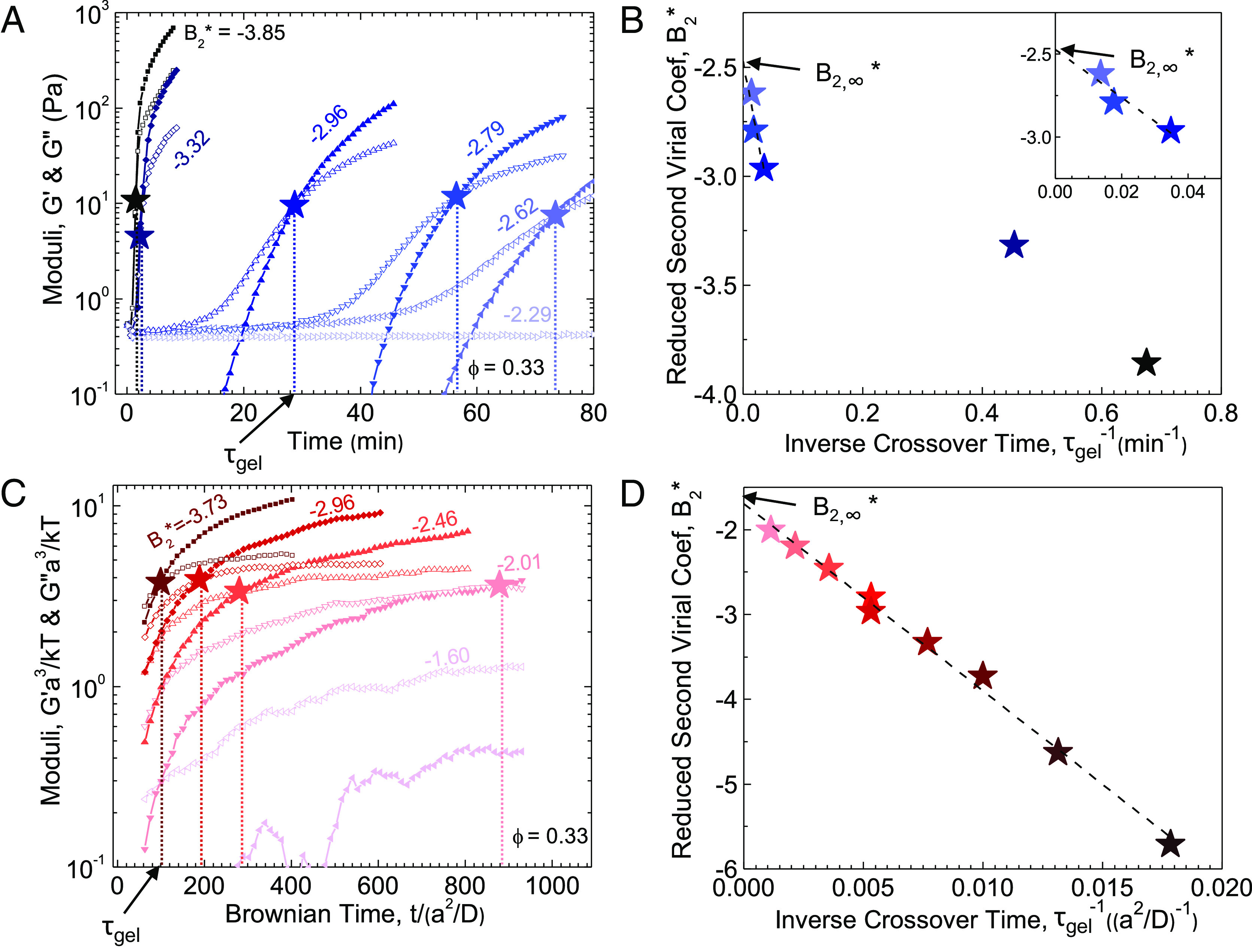
(*A*) Experimental linear viscoelastic responses, *G*′ (closed symbols) and *G*″ (open symbols), for samples with ϕ=0.33 after being quenched from the fluid phase at 20 ° *C*, $B_{2}^{*}=-0.33,$ to the final quench depths indicated in the legend. The time where *G*′=*G*″, $\tau_{\mathrm{gel}}$, is indicated with a star for each quench depth. (*B*) $B_{2}^{*}$ vs. the inverse cross-over time, $\tau_{\mathrm{gel}}^{-1}$, extracted for each temperature quench in (*A*). A linear extrapolation to $\tau_{\mathrm{gel}}^{-1}\to 0$ of the three shallowest quenches is performed (black dashed line) to determine the minimum quench depth necessary to form a gel, ${B_{2,\infty }}^{*}$. The inset shows a magnified view of the extrapolated data. (*C*) Linear viscoelastic responses from Morse simulations for ϕ=0.3. (*D*) $B_{2}^{*}$ vs. the inverse cross-over time, $\tau_{\mathrm{gel}}^{-1}$, extracted for each temperature quench in (*C*). Linear extrapolation of these data (black dashed line) gives ${B_{2,\infty }}^{*}$. For ease of viewing, only a subset of the quench depths used to construct (*D*) is shown in (*C*).

In our proposed method, SAOS measurements of the elastic (*G*′) and viscous moduli (*G*″) at fixed oscillation frequency and amplitude are monitored during fast temperature quenches (∼30 °C/min in experiments, and instantaneously in simulations) at a particular *ϕ* from the fluid state to a range of quench depths, $B_{2}^{*}$ (details given in *SI Appendix*). Once the quench is finished, samples are aged until either a dominant elastic response is observed (*G*′> *G*″) or a predetermined cutoff time is reached. In experiments, this was 5 h, after which sample evaporation can affect the observed behavior. In simulations, this was 1,000 *a*^2^/*D*, where *D* is the particle diffusivity, chosen to balance the need for a large system size with its relative computational expense. For quenches achieving a dominant elastic response, the time where *G*′=*G*″ denotes the apparent quench-dependent gel time *τ*_*gel*_. Example data are shown in Fig. 2*A* for the experimental system. As expected, *τ*_*gel*_ is quench-dependent, and progressively shallower quenches produce increases in *τ*_*gel*_ until solidification is no longer observed before the test is ended. In principle, as the system is quenched closer and closer to the gel point, the required gel time will diverge to infinity. To estimate this threshold, $B_{2}^{*}$ is plotted versus $\tau_{\mathrm{gel}}^{-1}$, and a linear extrapolation is performed to $\tau_{\mathrm{gel}}^{-1}\to 0$. Thus, we assume an asymptotic exponential divergence of the gel time as one approaches the minimal gel point. An example of this extrapolation for the experiments is shown in Fig. 2*B*. The extrapolated gel point, *B*_2, ∞_^*^, i.e., is the value of $B_{2}^{*}$ where *τ*_*gel*_ → ∞. This same method can be applied to the simulation systems (Fig. 2 *C* and *D*).

While this method can be applied to both experiments and simulations, different qualitative behavior is observed between the two (Fig. 2). Whereas experiments show two distinctly sloped regions of $B_{2}^{*}$ vs $\tau_{\mathrm{gel}}^{-1}$, simulated $B_{2}^{*}$ are linear over the complete range investigated. One simple explanation is that the timescales investigated in simulations are potentially much shorter than those in the experiments. Furthermore, for sufficiently deep quenches in experiments, $\tau_{\mathrm{gel}}^{-1}$ becomes comparable to the quench rate. This could result in the two regimes that we see in experiment, where for deep quenches (large $\tau_{\mathrm{gel}}^{-1}$) gelation occurs during the temperature ramp. By contrast, only one regime is observed in the simulations because the ramp is instantaneous and is fast relative to gelation for both shallow and deep quenches.

**Fig. 3.**
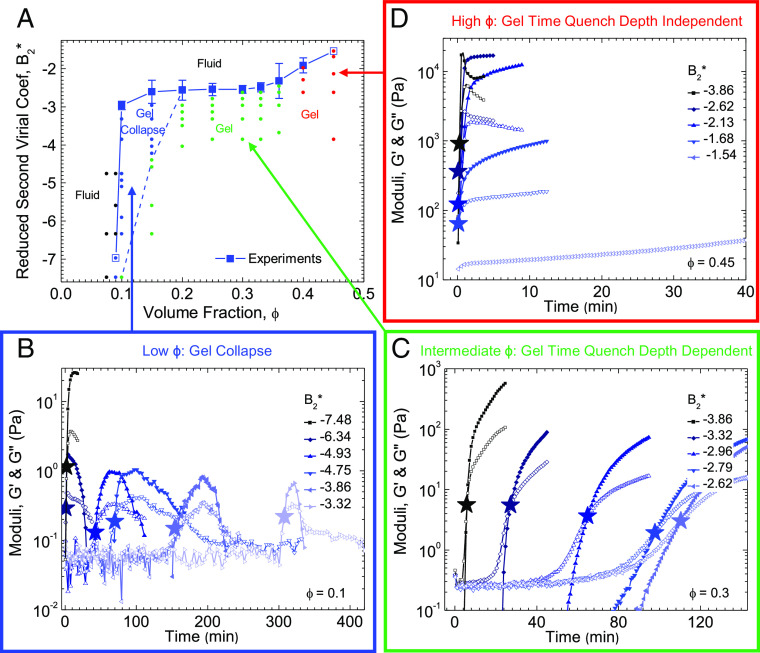
(*A*) Gel line for experiments (solid blue line) was determined by applying the extrapolation method from Fig. 2 for 0.1 ≤ *ϕ* ≤ 0.4 (solid blue squares) while the shallowest quench at which persistent cross-over occurred was used for *ϕ* = 0.09 and *ϕ* = 0.45 (open blue squares). The gel line has three distinctly sloped regions, low *ϕ* (positively sloped), intermediate *ϕ* (horizontal), and high *ϕ* (positively sloped). Distinct rheological responses are seen for quenches into the different *ϕ* regions of the gel line, gel collapse at low *ϕ* (blue points), gelation with quench depth dependent gel time at intermediate *ϕ* (green points), and nearly instantaneous gelation with quench depth independent gel time at high *ϕ* (red points). Quenches above and to the left of the gel line, in the fluid region (black points), show a dominantly viscous response over all time. The dashed blue line demarcates the transition from the gel collapse region to the persistent gel region. (*B*)–(*D*) Experimental linear viscoelastic responses, *G*′ (closed symbols) and *G*″ (open symbols), for *ϕ* = 0.1 (low *ϕ*), 0.3 (intermediate *ϕ*), 0.45 (high *ϕ*) samples after being quenched from *B*_2_^*^ = −0.33 to the final temperatures indicated in each graph legend. See supplemental information (*SI Appendix*, Fig. S3*A*) for the plot of $B_{2}^{*}$ vs 1/*τ*_*gel*_ for all *ϕ* used to determine the gel line in (*A*). Error bars in panel (*A*) represent the error in the y-intercept value from the linear extrapolations of curves in *SI Appendix*, Fig. S3*A*).

To determine the location of the gel line within the colloidal phase diagram, the process of Fig. 2 is carried out for multiple *ϕ* (Fig. 3 for experiments, and *SI Appendix*, Figs. S1 and S2 for the Morse and 2Y potentials, respectively). The results indicate three distinct regions of *ϕ*-dependence (*SI Appendix*, Fig. S3). At intermediate *ϕ* (green shaded region in *SI Appendix*, Fig. S3), the location of the gel line *B*_2, ∞_^*^(*ϕ*) appears to be insensitive to changes in *ϕ*. For low *ϕ* (blue shaded region in *SI Appendix*, Fig. S3), a steep dependence of $\tau_{\mathrm{gel}}^{-1}$ on *B*_2_^*^ makes it infeasible to extrapolate to $\tau_{\mathrm{gel}}^{-1}\to 0$. Instead, the location of the gel line at low *ϕ* is approximated as the shallowest quench that resulted in an elastic response. Conversely, at high *ϕ* (*SI Appendix*, Fig. S3, red shaded region), cross-overs in the rheology were observed at shorter times (higher $\tau_{\mathrm{gel}}^{-1}$). As $B_{2}^{*}$ is made more negative, cross-overs were no longer resolvable in the rheology because solidification occurred within the first oscillation cycle making it also infeasible to extrapolate for some high *ϕ*. Hence, to determine the location of the gel line at these *ϕ*, the shallowest quench that resulted in an elastic response was used to approximate the location of the gel line.

In summary, applying the method of Fig. 2 over a range of *ϕ* for the experimental system results in the gel line shown in 3 distinct regimes of gelation (Fig. 3*A*): It is steeply sloped in the low and high *ϕ* regions, but at intermediate *ϕ*, it is horizontal indicating that arrest in this region is insensitive to the initial *ϕ*. The gel lines for the Morse and 2Y potentials (obtained using the same method) exhibit the same 3 regimes, revealing gelation behavior that is insensitive to the detailed interaction potential shape. The location of the gel line determined from the above method is also insensitive to the applied SAOS oscillation frequency (*SI Appendix*, Text and Fig. S4).

Interestingly, the time evolution of viscoelasticity at quenches near the gel line are qualitatively different in these three regimes (Fig. 3 *B*–*D*). At low *ϕ* (blue points in panel (*A*) with characteristic rheology in panel (*B*)), quenches below the gel line initially produce a gel at short times. However, given enough time the elastic modulus begins to decrease, indicating gel rupture and eventually collapse. These transient gelling states have viscoelastic moduli that are orders of magnitude lower than those at higher *ϕ*, suggesting that although samples at a sufficiently low *ϕ* nominally contain enough particles to form a percolated network, phase separation can proceed and eventually rupture the gel due to its softness. This was visually confirmed using optical microscopy of gels formed in the experimental system (*SI Appendix*, Figs. S5 and S6). Similar gel collapse has been observed at low *ϕ* in depletion attraction systems (33, 65). Different behavior is observed at low *ϕ* for quenches much deeper than the gel line (*B*_2_^*^ ≥ −7.48 for *ϕ* = 0.1, black curve in panel *B*), where gels persist over observable time scales. We posit that the transition from gel collapse to persistent gels, marked in Fig. 3 with the dashed blue line, is set by the conditions where the yield stress of the initial gel structure can no longer resist gravitational and osmotic stresses due to phase separation, consistent with recent simulations that revealed the osmotic pressure difference between the dense gel strands and coexisting dilute phase to provide the driving force for collapse (63) (*SI Appendix* for further details).

At intermediate *ϕ* (green points in panel (*A*) with characteristic rheological behavior shown in panel (*C*)), quenches below the gel line produce quench-dependent gel times that increase as the quench depth is decreased. By contrast, at high *ϕ* red points in panel (*A*) with characteristic rheological behavior shown in panel (*D*), the gel time is invariant to quench depth, and gelation occurs within the first few oscillation cycles (nearly instantaneous) for quenches below the gel line. Similar behavior is observed in the simulation systems (*SI Appendix*, Figs. S1 and S2 for the Morse and 2Y, respectively), with the exception that gel collapse is not observed at low *ϕ*, as simulations are carried out without the effects of gravity. Prior simulations of gravitational collapse (63) established that although the osmotic stresses associated with phase separation are sufficient to explain the occurrence of gel collapse, gravity is often necessary to aid in initiating the collapse process.

### Thresholds for percolation, phase separation, and glassy arrest.

As previously speculated for other systems (33, 39), we hypothesize that the three regimes of the gel line arise from an interplay of percolation, phase separation, and glassy arrest. To test this hypothesis, we first identify the equilibrium phase boundaries and percolation lines for the different systems to compare with the observed gel lines. We illustrate the approach using the 2Y simulation system—the same approach was used for the Morse potential (*SI Appendix*). In experiments, the phase boundary and percolation line (Fig. 1*B*) were determined from previous predictions for the SW fluid (66–68) (*SI Appendix*, Fig. S6*C*).

**Fig. 4.**
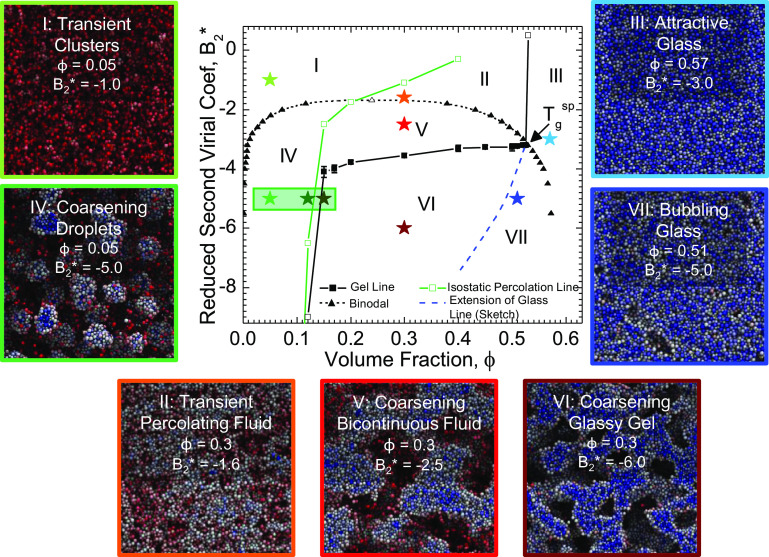
Equilibrium and nonequilibrium phase boundaries determined via dynamic simulation for the 2Y potential. The phase envelope was determined for the 2Y using the coexistence simulation method of Statt and Panagiotopoulos (70) (closed black triangles). The vapor-liquid critical point is indicated with an open black triangle and was determined from fitting the coexistence points following the method of Statt and Panagiotopoulos (with fit shown as the dashed black line). The isostatic percolation line (solid green line with square symbols) was constructed by analyzing particle snapshots using the method of Tsurusawa and Tanaka (9). An example of this analysis is shown for *B*_2_^*^ = −5.0 in *SI Appendix*, Fig. S12 with the location where snapshots were taken indicated with the green box. The resulting phase envelope and percolation line are overlaid with the gel line (solid black line with square symbols) determined via protocol of Fig. 2. The blue dashed line is a sketch of the attractive glass line extending into the phase instability region. These lines break the phase diagram into seven regions, each with unique morphology as shown in the particle snapshots. Stars on the diagram indicate where the snapshots in each region were taken. Particles in snapshots are colored according to the number of contacts for each particle, *N*_*C*_, as follows: Freely diffusing particles are colored red, particles having few contacts are colored white and particles with many contacts are colored blue.

In simulations, the equilibrium phase boundary (black dashed line with triangle symbols in Fig. 4) was determined by adapting the method of Statt and Panagiotopoulos (70). Briefly, a simulation was initialized with coexisting dense and dilute regions, instantaneously quenched, and then aged until equilibrium between the phases was achieved. *ϕ* was then calculated in each phase to determine the location of the phase boundary (*SI Appendix*, Text and Fig. S7). The results were confirmed by collecting simulated system snapshots at various *ϕ* and *B*_2_^*^ under quenching from an initially uniform density to visually verify 1) the quench depth corresponding to the critical point, and 2) the location of the intersection of the phase boundary with the gel line (*SI Appendix*, Figs. S8 and S10 for the 2Y and Morse potentials, respectively).

The number of nearest neighbor particle contacts, *N*_*C*_, was also tracked to determine the probability distribution of contacts, *P*(*N*_*C*_). The time evolution of *P*(*N*_*C*_) exhibits distinct behavior above and below the fluid–fluid critical point (*SI Appendix*, Figs. S9 and S11 for the 2Y and Morse potentials, respectively). Similarly, distinct *P*(*N*_*C*_) evolution is also observed above and below the gel line (*SI Appendix* for details). Ultimately, the phase boundaries determined from analysis of *P*(*N*_*C*_) are in excellent agreement with those determined from the coexistence simulations, cross-validating the two methods of inferring the equilibrium phase boundary.

To identify the percolation line, we adopt a local criterion of rigidity as defined using Maxwell’s rule for isostaticity (69). The isostatic percolation line (green line in Fig. 4) was constructed following the method of Tsurusawa and Tanaka (9). Briefly, we detected combinations of *ϕ* and *B*_2_^*^ that correspond to the emergence of percolating clusters of isostatic particles (particles forming at least six contacts with neighboring particles, *N*_*C*_ ≥ 6). The transition from multiple large clusters to a single dominant percolating cluster (containing more than 99% of all isostatic particles) is a more stringent condition and marks the isostatic percolation transition. Details on how the percolation line was constructed can be found in *SI Appendix*, text with particle snapshots shown in *SI Appendix*, Figs. S12 and S13. We note that isostaticity is only one possible criterion for determining percolation, with rigidity percolation (based on a global analysis of network structure) being a common alternative (24). The potential consequences of this choice will be discussed later.

### Dependence of the state diagram on interparticle potential.

**Fig. 5.**
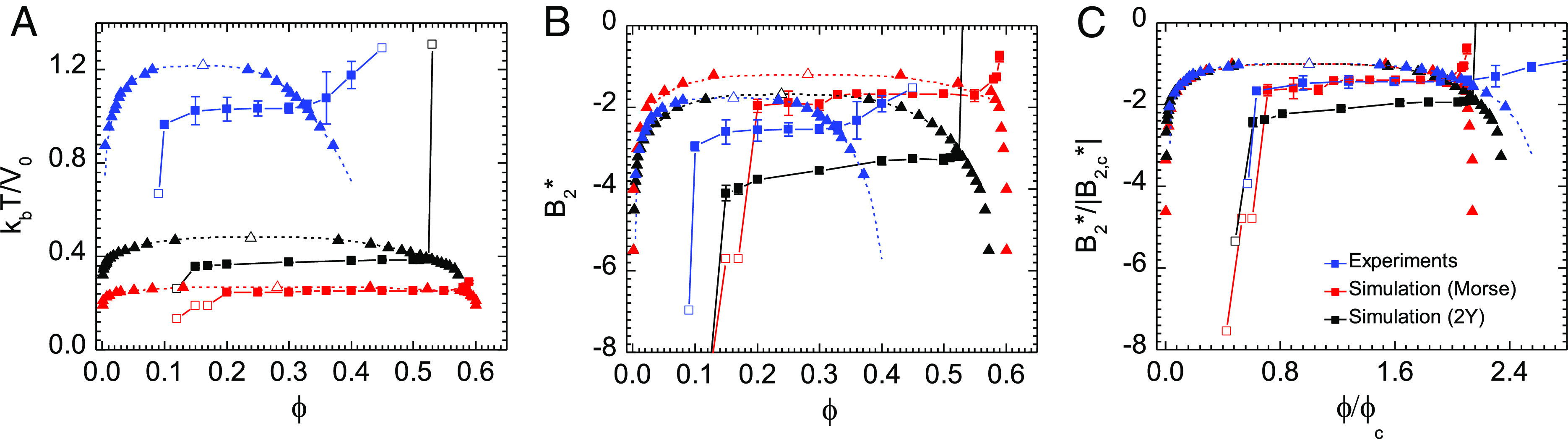
Colloidal phase diagrams with inscribed gel lines for experiments (solid blue line with squares), Morse simulations (solid red line with squares) and Two-Yukawa simulations (solid black line with squares) shown with their respective phase envelopes (dashed blue line with triangles for square-well phase envelope, dashed red line with triangles for Morse envelope and dashed black line with triangles for Two-Yukawa envelope) (*A*) *k*_*b*_*T*/*V*_0_ vs. *ϕ* representation (*B*) *B*_2_^*^ vs. *ϕ* representation (*C*) *B*_2_^*^/|*B*_2, c_^*^| vs *ϕ*/*ϕ*_*c*_ representation: Data from (*B*) have been collapsed by scaling each gel line and phase envelope pair by their respective critical values, $B_{2,c}^{*}$ (−1.77, −1.68, −1.19 for SW, 2Y, and Morse, respectively ) and *ϕ*_*c*_ (0.16, 0.25, 0.28 for SW, 2Y, and Morse, respectively).

When comparing the location of the minimal gel line to other important transitions (phase boundary, percolation line, glass line) for the three experimental and computational model systems, we find similar qualitative behavior regardless of the shape and range of the potential (Fig. 5). To further interrogate this similarity, we examine three different representations of the phase diagram: A) *k*_*b*_*T*/*V*_0_ vs *ϕ* where *V*_0_ is the potential well-depth, B) *B*_2_^*^ vs *ϕ*, and C) *B*_2_^*^/|*B*_2, Crit_^*^| vs *ϕ*/*ϕ*_*c*_ where (*ϕ*_*c*_, *B*_2, c_^*^) denotes the vapor-liquid critical point. In all cases, the same three-region behavior of the gel line reported in Fig. 3 is observed. When the state diagrams are compared in *k*_*b*_*T*/*V*_0_ space, we observe that both the equilibrium phase boundaries and the nonequilibrium gel lines shift monotonically up and to the left with increasing interaction potential range in a way that is consistent with previous studies on how potential range shifts equilibrium phase boundaries (66, 68), percolation lines (67), and attractive glass lines (32, 36).

One might wonder whether the precise locations of the two important features of the gel line—the isostatic percolation line and *T*_*g*_^*sp*^—could be quantitatively mapped in a way that is insensitive to the shape of the potential. Some studies suggested that an extended law of corresponding states (ELCS) can collapse these features for short-ranged attractive colloidal systems when data are plotted using the reduced second virial coefficient (12, 41, 71). However, others claimed that a different parameter, such as the potential well depth, is needed to collapse nonequilibrium behavior (26). From Fig. 5 it is evident that for the experimental and simulated potentials tested in this work, neither *B*_2_^*^ nor *k*_*b*_*T*/*V*_0_ collapse the equilibrium and nonequilibrium lines. This is not unexpected because the ELCS is not believed to hold for systems with longer-ranged potentials like those investigated here. Alternatively, we identified that rescaling *ϕ* and *B*_2_^*^ by their respective critical point values, *ϕ*_*c*_ and *B*_2, c_^*^, respectively, approximately collapses the phase envelopes both vertically and horizontally (Fig. 5*C*). This scaling also approximately collapses the vertical and horizontal locations of the gel lines. Although this collapse is not exact, this observation could allow one to predict the location of the gel line for systems characterized by different interparticle potentials knowing only the equilibrium fluid–fluid critical point (*SI Appendix* for further discussion).

### Interplay of gelation with percolation, phase separation, and glassy arrest.

We illustrate the relation of the gel line to the previously proposed mechanisms of gelation using simulations involving the 2Y potential. Notably, the 2Y gel line (solid black line in Fig. 4) changes slope at two state points that coincide closely with the value of *B*_2_^*^ corresponding to *T*_*g*_^*sp*^: 1) at *ϕ* ∼ 0.15, where the isostatic percolation line crosses *T*_*g*_^*sp*^, and 2) at *ϕ* = 0.52 and *B*_2_^*^ = −3.2, where the *T*_*g*_^*sp*^ point itself resides. This suggests that percolation, phase separation, and the glass transition all play a significant role in controlling where gelled states appear. This same qualitative behavior is observed for all of the systems studied (Fig. 5).

The intersection of the gel line with the percolation line and equilibrium phase boundary splits the phase diagram into seven distinct regions (Fig. 4), and particle snapshots elucidate the morphologies observed in each region for the in silico 2Y system. In region I, interparticle attractions cause the formation of equilibrium clusters, whose density is too low and the particle attractions too weak for clusters to percolate or for phase separation to occur, and therefore no elastic response is observed. In region II, the particle density is sufficient to form percolated networks, but the lifetime of these networks is short and attractions too weak to induce phase separation, resulting in an equilibrium transient network with mechanical response corresponding to a viscoelastic liquid. In region III, an attractive glass is formed essentially instantaneously upon quenching due to the high particle density and slow dynamics. In region IV, interparticle attractions are strong enough to induce phase separation, but the density is too low to form percolated structures, resulting in small droplets of particles. In region V, attractions are strong enough to induce phase separation. Although the density is sufficiently high for percolated structures to form, the bond strength is insufficiently “cold” to produce an elastically dominant response at low frequencies; thus, as in region IV, phase separation can proceed to completion. In region VI, gels form as bonds are sufficiently “cold” and dense to cause phase separation, followed by structural arrest via formation of an attractive glass in the strands when the density of the dense phase intersects the glass line within the region of phase instability (dashed blue line). In region VII, an attractive glass also forms similar to region III. Whereas in region III a homogeneous glass forms from the homogeneous fluid “phase”, in region VII glassy behavior hinders phase separation, although over time, particle free voids or “bubbles” form as the system attempts to phase separate, producing a heterogeneous glass. However, this coarsening densifies the continuous particle network, further impeding phase separation so that it arrests at a very early stage.

Similar morphologies have been observed in other systems. For example, Sedgwick et al. (39) reported a “spherical bead” morphology similar to the “coarsening droplet” structures observed in this work. Bead-like morphologies were also identified in theoretical work by Cates et al. (13). Additionally, prior work by Gibaud et al. (26) and Cates et al. argued for the formation of a homogeneous attractive glass morphology inside the phase boundary. Although we observe a homogeneous arrested state at early age times after quenching, as the system ages phase separation occurs, and we observe formation of a heterogenous glass with particle-free voids or “bubbles”. Interestingly, this age-dependent transition between an initially homogeneous glass and a “bubbling glass,” which is apparent in both the evolution and structure and rheology for quenches into region VII (*SI Appendix*, Fig. S14), was not reported in these prior works.

To reconcile this behavior with the previously proposed mechanisms of gelation, we highlight a potentially surprising observation: In each of the experimental and computational systems studied, the gel line lies significantly below the isostatic percolation line for a large range of volume fractions (typically above *ϕ* ∼ 0.10). In other words, although isostatic percolation appears to be a necessary condition for solidification—in agreement with recent findings in other gelling systems (9), it is clearly not sufficient for gelation over a large range of thermodynamic state space. One may wonder whether this finding is sensitive to the definition used for the percolation threshold. For example, Zhang et al. (24) alternatively identified a correlated rigidity percolation threshold in thermally fluctuating 2D gels using the pebble-game algorithm, and proposed that gelation occurs when the gel spends a majority of configurations adopting a rigid structure. Such a definition applied to the present work should reconcile at least some of the observed discrepancy between the percolation line and the gel line. However, this could not be tested in the present simulations because the pebble- game cannot be readily implemented in the large, three-dimensional systems studied here. Regardless, we emphasize that current definitions of percolation (e.g., isostatic connectedness or instantaneous rigidity) do not test for the persistence of a particular rigid structure in time under the influence of thermal fluctuations. Such persistence is necessary for solidification, as shown in the present study and achieved in the long-time structures of Zhang et al. (24). That is, for percolation to successfully form a solid, bonds must also be either sufficiently “cold” so as to be essentially permanent over observable time scales, or sufficiently “crowded” that the collective dynamics of the system become arrested—thus producing a percolating structure that persists in time. Even so, a percolated gel may still be susceptible to osmotic collapse, as we observe in experiment in a region of quenching just to the right of the isostatic percolation line (Fig. 3).

We also find that phase separation is by itself insufficient to form a gel. Specifically, there is a significant region of state space between the vapor-liquid binodal and the gel line for all systems studied (Region V in Fig. 4) where gelation is not observed. This is in contrast with the findings of Lu et al. (12) on gels forming by short-ranged depletion attractions, where phase separation (and in particular, spinodal decomposition) was claimed to be both necessary and sufficient for gelation. Rather, we find that whether phase separation is necessary to form a gel depends in a complex manner on the depth of quenching. For example, we expect that an asymptotically deep quench into the phase instability region in Fig. 4 will gel sufficiently fast and with sufficient elasticity to completely preclude phase separation, and produce gels either resembling those formed at lower *ϕ* by diffusion-limited cluster aggregation (DLCA) (13, 72–74) or at higher *ϕ* by glassy arrest (region VII). This is consistent with some previous scattering measurements on the experimental nanoemulsion system that revealed fractal-like gel microstructure for deep quenches below the low-*ϕ* branch of the gel line (16). In this case, phase separation is not necessary to form a gel. By contrast, for milder quenches into region VI, phase separation is necessary to produce a gel. Here gels form through phase separation when the density of the colloid-rich phase hits the attractive glass line (dashed blue line in Fig. 4), leading to the formation of an attractive glass in the particle rich strands in a process typically called “arrested phase separation” (10, 12, 26, 27, 30, 75).

From the situation just described, we see that glassy arrest can also be a necessary condition for gelation. At high *ϕ* outside the equilibrium vapor-liquid binodal, the attractive glass line clearly sets the conditions for gelation (Region III in Fig. 4). Furthermore, below the binodal, gels only form for quenches below *T*_*g*_^*sp*^. Previous work (26, 27, 30, 75) also showed that a critical quench depth inside the binodal was necessary for gelation corresponding to *T*_*g*_^*sp*^ (also named *T*_*a*_ in some studies). However, these past works did not resolve the exact shape of the gel line over all *ϕ* within the region of phase instability. Our results show that for all *ϕ* above the percolation threshold, the gel line is roughly horizontal with *B*_2_^*^ = *T*_*g*_^*sp*^. As previously mentioned, this suggests that gels form below this line (Region VI in Fig. 4) through phase separation once the strands of the emerging dense phase reach a local density equal to the location of the glass line within the region of phase instability (dotted blue line in Fig. 4), at which point glassy arrest is believed to occur within the dense phase strands (10, 12, 26, 27, 30, 75). While the exact shape of the glass line in the phase instability region could not be verified in this work, this concept is consistent with what we observe in simulations for quenches into Region VI (*SI Appendix*, Fig. S15), where the emergence of elasticity only occurs once a peak at *N*_*c*_ = 9 is reached in the *P*(*N*_*c*_) distribution, consistent with the formation of an attractive glass in the dense phase (7).

It is interesting to contemplate what sets the transition just described between a “crowded” phase-separated gel at mild quenches below the gel line (where phase separation is necessary for gelation) and a “cold” fractal- or bubbling glass-like gel at deep quenches (where it is not). Cates et al. (13) speculated that this transition is uniquely selected by two factors—i) the initial *ϕ* from which the system is quenched, and ii) the rate of quenching. However, our results (Fig. 3) reveal an intriguing possibility—that this transition is determined by not only the quench depth (instead of or in addition to the quench rate), but more importantly varies in time after the quench is executed. Specifically, a system may initially form a “cold” gel at short times for sufficiently deep quenches by a DLCA-type process. However, as discussed above, whether this gel morphology persists at long age times after it is formed, or instead transforms into a “crowded,” phase-separated gel, will be determined through a complex interplay between the kinetics of phase separation and the potential for osmotically driven collapse of the nascent gel. This phenomenology is consistent with what is observed experimentally in the blue region of Fig. 3, where gels initially forming at low *ϕ* experience collapse and observable phase separation (*SI Appendix*, Figs. S5 and S6).

One may wonder whether this same framework can be used to reconcile the behavior of other well-studied experimental systems. The systems with behavior most akin to that observed here are the protein systems studied by Schurtenberger et al. (26, 27) and Sedgwick et al. (39), as well as the depletion system of Harich et al. (33), which have all proposed piecewise straight gel lines in the region of fluid–fluid phase coexistence. While the gel lines from those works follow the same general shape we report, there are a number of notable differences. For example, in the work by Schurtenberger et al., the low *ϕ* behavior we report was not resolved, and so no comparison with the percolation threshold could be made. Similarly, Sedgwick et al. did not resolve the gel line outside the equilibrium phase boundary. Lastly, discrepancies between the experimentally and theoretically determined gel lines from Harich et al. prevent a detailed interpretation using the framework of competing gelation mechanisms we argue for in the present work. Despite these differences, the underlying combination of mechanisms that set gelation (percolation, phase separation, and glass transition) proposed in these works agree qualitatively with what we observe, suggesting some level of transferability of the picture of gelation illustrated by Fig. 4.

This generality suggests that for systems characterized by interactions with short- or moderate-range attraction, gelation behavior appears to be insensitive to the system chemistry or the exact form of the attractive potential. However, in systems where particles bond through more complex mechanisms such as frictional adhesion, bending stiffness, or patchy interactions, other behavior may be observed. For example, in the system of thermoresponsive octadecyl-coated silica studied extensively by multiple groups (15, 47, 48), it was concluded that gels always form by percolation, with a gel line that follows the percolation line throughout the supercritical region of the phase diagram. In the present framework, this could correspond to a case where the transition between a persistent solid gel to a temporary viscoelastic network lies at quenches above the equilibrium phase boundary. We note that such a state may be aided in that system by bonds with frictional adhesion or bending stiffness that is not present in other experimental systems, such as those studied here. Alternatively, in the case of colloids with polymer-induced depletion attractions (12), it was initially observed that the gel line follows the equilibrium vapor-liquid phase boundary. Although the present work clearly shows that phase separation is not a sufficient condition for gel formation, such a situation could occur when the location of *T*_*g*_^*sp*^ falls very close to the equilibrium vapor-liquid critical point. Indeed, for many systems with short-range attraction, the shape of the vapor-liquid binodal is very flat in the vicinity of the critical point, and so any experimental uncertainty in its location or in the location of *T*_*g*_^*sp*^ may lead one to infer that the two are identical. Nevertheless, within the framework considered here, persistent gels can only form for quenches below *T*_*g*_^*sp*^ in such a system.

### Time-dependent behavior and the potential for thermal processing.

The juxtaposition of quench- and age-dependent gelation kinetics (Fig. 2) with our colloidal state diagram (Fig. 4) provides insights into the potential for thermal processing of structure and properties of an emergent colloidal gel. As originally suggested by Cates et al. (13), in the present thermoresponsive systems one can controllably move through multiple regions of the state diagram in time by manipulating temperature, and the rate (either step-wise or continuous) at which the thermal quench is executed will impact the final structure and material properties. In this regard, precise knowledge of the gel point (i.e., the boundary between regions V and VI in Fig. 4) allows one to control, for a given quench path, the amount of time spent in region V where phase separation is allowed to proceed uninterrupted by the onset of solidification. Likewise, for deeper quenches into region VII, one can control the amount of time where viscoelastic phase separation - which still proceeds but is now frustrated by the dominant elastic response of the system (35) - is allowed to dominate the coarsening of the emergent gel structure before the onset of deep glassy arrest.

**Fig. 6.**
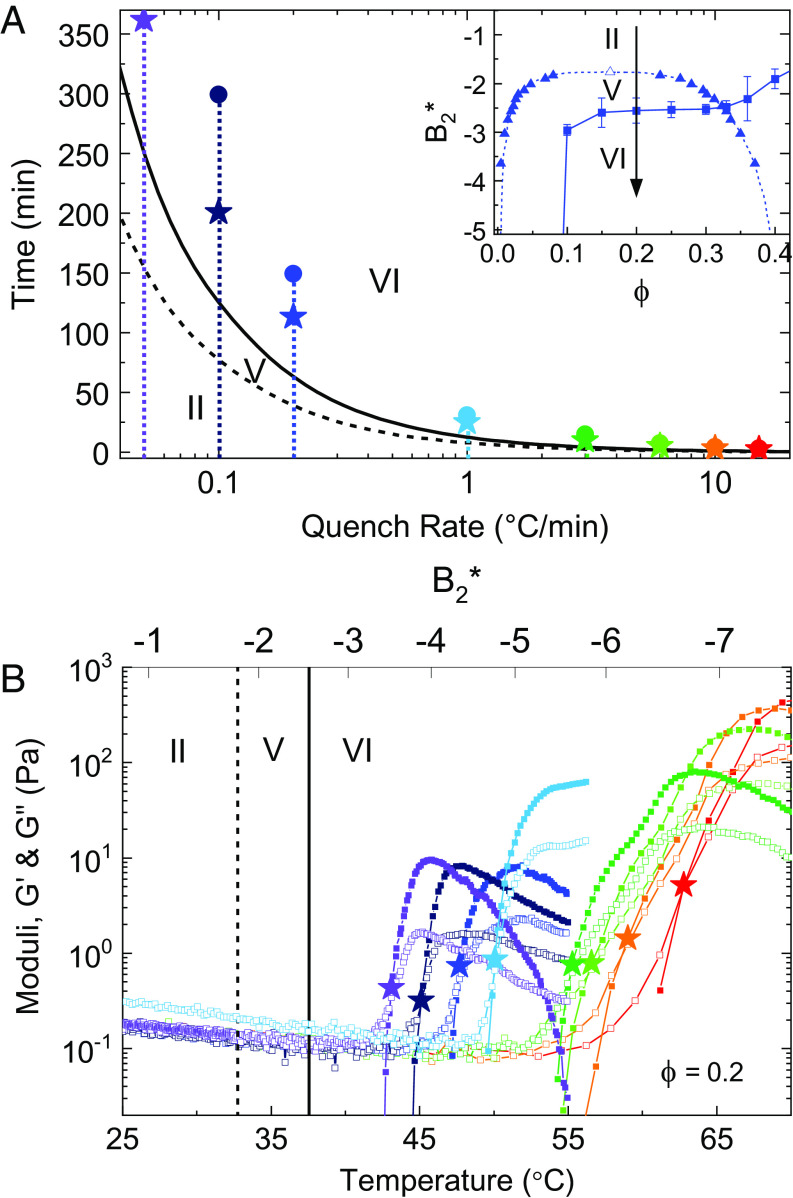
The effect of quench rate on gel formation and aging for the experimental system at *ϕ* = 0.2. Temperature ramps start at *T* = 25 ° *C* (*B*_2_^*^ = −0.80, region II in Fig. 4) and ramp linearly at different rates (in °*C*/*min*). The inset in (*A*) shows the executed quench (black arrow) through the experimental phase diagram. (*A*) Time spent in regions II, V, and VI (lines) depends on the quench rate. Colored points indicate the elapsed time of the temperature ramp (circles) and the approximate solidification time (where *G*′=*G*″, stars) for each quench rate. (*B*) Evolution of linear viscoelastic moduli *G*′ (closed symbols) and *G*″ (open symbols) for each quench rate. The gelation temperature for each quench rate is indicated with a star. A linear temperature ramp represents a nonlinear ramp in *B*_2_^*^, so a nonlinear *B*_2_^*^ axis has been added to the *Top* of the plot.

For example, this work has so far explored gels that form after instantaneous quenching from region II to region VI. However, if instead one performs a sufficiently slow quench along the same path, we anticipate that the sample will macroscopically phase separate in region V before gelation, thus producing a softer gel or no long-lived gel at all. This idea is demonstrated in Fig. 6 for the experimental system for an initial volume fraction of *ϕ* = 0.2 (for *ϕ* = 0.33 *SI Appendix*, Fig. S16), where samples differentially quenched at different rates experience different amounts of time in regions II, V, and VI, and as a result exhibit aging kinetics and long-time gel moduli that vary by two orders of magnitude depending on the quench rate. In Fig. 6, the spatiotemporal trajectory of each quench is shown in (*A*) along with the corresponding evolution of viscoelastic moduli in (*B*).

It is not surprising that the observed quench rate-dependence was unreported in previous studies, since for many experimental systems quenching is obtained by mixing components, prohibiting controlled quenches without careful experimental design (9). Moreover, the large quench-dependent changes in final gel properties observed here were not observed in previous work assessing the potential for step-wise quenches to modulate the long-time mechanical properties of gels in the same experimental system (55). The results here provide a potential explanation, in that step-wise quenching was performed sufficiently fast so as to miss the important window of quench rates whereby the rate of the quench is slow relative to the rate of phase separation (Fig. 6). Consequently, the conditions of quench rate that produce differentially large changes in gel structure and elasticity in region VI will be highly sensitive to the amount of time spent in region V during the quenching, and to the complex microdynamics of phase separation known to occur in such a state (35).

Confirming these observations using simulations would require a significant effort to simulate the long age times, and to account for the more subtle temperature-dependence of collective particle-scale dynamics in the experimental system (50) which are known to influence gelation kinetics (76). We hope that the current experiments will inspire such refined simulations in future work. More broadly, while a thorough investigation of the impact of the detailed quench protocol on gel structure and rheology is beyond the current scope, the present results encourage the exploration of this frontier for sculpting colloidal gel structure and mechanics.

## Conclusion

Developing a complete understanding for the mechanisms of gelation in colloidal systems is crucial for designing soft colloidal materials, but this endeavor has been made difficult by the lack of robust methods for determining the location of arrested states in a phase diagram. To address this problem, we presented a new method for determining the minimal gelation threshold for quench-controlled colloidal systems in experiment and simulation using an extrapolation of differential quench measurements. The gel lines determined using this method exhibit three distinct regions for systems with different attractive potentials. A combination of detailed microstructural and rheological measurements reveals that these regimes are set by a complex interplay of percolation, phase separation, and glassy arrest. Importantly, we observe that neither isostatic percolation nor unstable phase separation by themselves is sufficient to produce a gel over the entire phase diagram. Nevertheless, we find that the transitions between regimes of gelation are set by two critical parameters - the isostatic percolation threshold and the intersection of the attractive glass transition with the equilibrium binodal. We showed that the equilibrium vapor-liquid critical point can be used to collapse the dependence of these features on the shape of the colloidal interaction potential, suggesting a means by which their location can be estimated for other model systems.

The complex behavior revealed by our study, in which an interplay of kinetic processes representing aggregation, coarsening of phase separation, and glassy dynamics that compete to form and restructure an emerging colloidal solid, leads to rich nonequilibrium phase behavior that holds significant potential for dynamically controlling the structure and properties of a nascent gel. This was demonstrated by identifying regions of the phase diagram (V and VI in Fig. 4) where a phase separating gel is neither too “cold” nor too “crowded,” such that its developing structure is highly susceptible to the kinetic path of quenching taken through these regions to a prolonged arrested state. Quench rate-dependent gelation experiments revealed that the “final” properties of a gel can be strongly manipulated by controlling the thermal trajectory through the region of phase instability before a deep quench into the glassy state. The results call for a theoretical framework to understand how the kinetics of quenching through the phase diagram impacts gel microstructure and phase mechanics. Moreover, they portend processing schemes for colloidal gels akin to thermal processing of metals and ceramics, in which complicated annealing, tempering, and quenching strategies that leverage the insights of the present work can be used to create colloidal solids with exceptional or unprecedented properties.

## Materials and Methods

### Experimental Methods.

Polydimethylsiloxane (PDMS, 5 cSt viscosity)-in-water nanoemulsions containing poly(ethylene glycol) diacrylate (PEGDA) were prepared by high-pressure homogenization according to previous methods (50) (*SI Appendix*, section 1.1 for details). Droplet sizes and polydispersity were measured using dynamic light scattering (DLS) according to previous methods (50) (*SI Appendix*, section 1.2 for details). Rheological measurements were performed according to previous methods (11, 14) (*SI Appendix*, section 1.4 for details). Optical microscopy was performed on an inverted visible light microscope with temperature control using a dual-Peltier stage according to previous methods (35).

### Simulation Methods.

We constructed our computational model system using the LAMMPS molecular dynamics package. Our computational model system comprises a suspension of nearly hard spheres suspended in an implicit solvent (*SI Appendix*, section 2.2 for details). Rheological measurements were performed according to previous methods (7, 58) (*SI Appendix*, section 2.3 for details). Methods to determine the percolation line and phase boundary were discussed briefly in *Results* and *Discussion* Section (*SI Appendix*, sections 2.4 and 2.5 for details).

## Supplementary Material

Appendix 01 (PDF)

## Data Availability

Primary numerical data for all figures in the main text and *SI Appendix* have been deposited in the Dryad Data Repository (77), and are available upon publication of this work.
