# Supplementary material for: Minimal conditions for solidification and thermal processing of colloidal gels
Source: Proc Natl Acad Sci U S A. 2023 Jun 12;120(25):e2215922120. doi: 10.1073/pnas.2215922120 (PMC10288545; doi:10.1073/pnas.2215922120)
Supplement: Supplementary file 1 — Appendix 01 (PDF) [file pnas.2215922120.sapp.pdf]

www.pnas.org

Supplementary Information for

Minimal conditions for solidification and thermal processing of colloidal gels

Scott M. Fenton, Poornima Padmanabhan, Brian K. Ryu, Tuan T.D. Nguyen, Roseanna N. Zia, and Matthew E. Helgeson

Matthew E. Helgeson.
E-mail: helgeson@ucsb.edu

This PDF file includes:

Supplementary text
Figs. S1 to S18
SI References

Supporting Information Text

1. Experimental System

1.1 Nanoemulsion Preparation. The experimental oil-in-water nanoemulsion system is comprised of a dispersed phase of poly-dimethylsiloxane (PDMS, viscosity=5 cSt) droplets and an aqueous continuous phase of sodium dodecyl sulfate (SDS) surfactant, poly(ethylene glycol) diacrylate bridging polymer (PEGDA, $M_n = 700 gmol^{-1}$), and deionized water. All chemicals were purchased from Sigma-Aldrich without further purification. Nanoemulsions of various dispersed phase volume fractions, ϕ, were made via homogenization or ultrasonication (for processing details see next paragraph). All nanoemulsion samples were prepared with a continuous phase polymer volume fraction of P=0.33 PEGDA. To ensure that the PEGDA concentration was uniform throughout the prepared samples (and thus that the interdroplet interactions were uniform), additional experiments (discussed in section 1.7) were carried out to quantify the concentration of polymer in the droplet rich and droplet poor phases formed during phase separation. In addition to ensuring that the polymer concentration was uniform, it was also critical to keep the final "free" surfactant concentration (the surfactant concentration in the bulk continuous phase) fixed to ensure that the repulsive electrostatic interactions from the charged SDS at the droplet interface were constant as a function of ϕ. To do this, the input SDS surfactant concentration was changed with ϕ to keep the final "free" surfactant concentration at 20mM for all ϕ. For $\phi = 0.2$, 0.3 and 0.4 this corresponds to total input SDS concentrations of 130, 180 and 240 mM SDS, respectively. The input SDS concentrations were determined using the work of Pangenkopp and Mason (1) who reported how SDS partitions between the PDMS droplet interface and the continuous phase. As can be seen from the reported values above, for smaller ϕ, a lower input surfactant concentration is needed to obtain a final "free" surfactant concentration of 20mM because less surfacant goes towards forming the droplet interface. The importance of the surfactant concentration can be seen by comparing the gelation transition reported in this work (where samples were prepared with the same final "free" surfactant concentration for all ϕ) with the transition reported by Hsiao(2) (where samples were prepared with the same input surfactant concentration). The gel line reported by Hsiao is oppositely sloped to the one reported in this work due to the significantly greater screening of repulsive electrostatic interactions at low ϕ.

The nanoemulsions, with the compositions described above, were prepared as follows: a crude emulsion was prepared by mixing the continuous phase (PEGDA, SDS, DI water) with a stir plate set at 700 rpm followed by dropwise addition of the dispersed phase (PDMS). High pressure homogenization of emulsions with $\phi = 0.1, 0.15, 0.2, 0.25, 0.3, 0.33$ were done using the Avestin Emulsiflex-C5 homogenizer operating at 15 kpsi. Between each pass the samples were cooled in ice and 12-16 passes were needed until the desired droplet sizes were achieved. The high volume fractions, $\phi = 0.36, 0.4, 0.45$, were processed using a Fisher Scientific Sonic Dismembrator Ultrasonic Processor instead of the homogenizer because higher ϕ samples gel during processing and clog the homogenizer valves. The samples were stirred continuously during ultrasonication processing using a stir plate and cooled using an ice bath. The droplet sizes were periodically checked during processing until the desired droplet size was achieved. After synthesis, the nanoemulsions were stored at $8^{\circ}C$ to slow droplet growth.

1.2 Droplet Size Characterization by Dynamic Light Scattering. Droplet sizes were measured during ultrasonication and homogenization using dynamic light scattering (DLS). DLS was done using a Brookhaven Instruments BI-200SM system equipped with a 532nm laser. Before measurements, $100\mu l$ of nanoemulsion were diluted in 3ml of DI water. Measurements were conducted at $20^{\circ}C$ at a scattering angle of 90°. All samples were prepared with similar size and polydispersity. The diameter of nanoemulsion samples are $48 \pm 4nm$ with polydispersity index (PDI) of 0.26 ± 0.03.

1.3 Characterizing Experimental Particle Interactions. The interaction that arises between nanoemulsion droplets from bridging by PEGDA oligomers was previously modeled by Helgeson et al. (3) using a square-well (SW) potential which was extracted from small angle neutron scattering (SANS) measurements of a dilute, $\phi = 0.01$, nanoemulsion sample. The resulting SW potential is shown in Fig. 1(b) and its functional form is defined below:

Square-Well:

$$\frac{V(r|T)}{k_bT} = \begin{cases} \infty, & r \leq 2a \\ -V_0(T)/k_bT, & 2a < r \leq 2a\lambda \\ 0, & r > 2a\lambda \end{cases} \quad [1]$$

where r is the center-to-center distance between particles of radius a, T is temperature, k_b is Boltzmann's constant, $V_0(T)/k_bT$ is the temperature dependent well depth and λ is the potential range. For the SW potential shown in Fig. 1(b) $\lambda = 1.5$, and is set by the extended end-to-end length of the PEGDA bridging polymer.

1.4 Rheological Characterization. To determine the gelation threshold for the experimental system, temperature quenches with aging were performed while the viscoelastic moduli, G' and G'', were monitored using small amplitude oscillatory shear (SAOS) rheological characterization performed on a TA Instruments ARG2 rheometer equipped with a 60mm, 2° upper-cone geometry and a Peltier bottom stage. Temperature quenches were performed with the fastest quench rate that the rheometer could obtain ($\approx 30^{\circ}C/min$) from $20^{\circ}C$ ($B_2^* = -0.33$) to the various final temperatures. SAOS was carried out with an applied oscillation frequency of $\omega = 10 rads^{-1}$ and an oscillation strain of $\gamma = 0.1\%$.

In addition to the quench measurements used above to determine the gelation threshold, temperature ramp experiments were also conducted on the ARG2 to evaluate how changing the ramp rate from the fluid phase (region II in Fig. S6(c)) into the gel region (region VI) impacts the resulting gels mechanical properties. G' (closed symbols) and G'' (open symbols) are

shown for several ramp rates for two colloidal volume fraction, $\phi = 0.2$ and $\phi = 0.33$ in Fig. S16. Temperature ramps start at $T = 25°C$ ($B_2^* = -0.80$, region II in the experimental state diagram) and ramp at different rates into the phase instability region (region V) before crossing the gelation line and entering region VI. During temperature ramps, SAOS was carried out at the same frequency and amplitude used for temperature quench experiments.

To determine the sensitivity of the gelation threshold found through the temperature quench experiments to the frequency used during SAOS, frequency sweeps were carried out at quenches above, at, and below the gelation threshold after samples had been aged for 2 hours (Fig. S4(b)). The strain amplitude was held fixed at $\gamma = 0.1\%$. For a detailed discussion of the frequency dependent behavior of the gelation threshold see section 4.

1.5 Investigating Gel Collapse Using Light Microscopy. As was briefly mentioned in the main text, gel collapse was observed in the experimental rheology taken at low ϕ. This behavior is discussed in more detail below. Unlike higher ϕ, where long lived gels formed upon quenching, at low ϕ, low modulus gels initially formed, but given enough time gel collapse occurred. An example of this behavior is shown for $\phi = 0.1$ in Fig. S5(a) for a quench to $B_2^* = -5.60$. This rheological behavior indicates that the particle density and attraction strength are just sufficient to form a elastic percolating network. However, the network that forms is too weak to resist further phase separation, so structural coarsening continues until the gel collapses and macroscopically phase separates. To confirm this, we observed the evolution of structure during the gelation and subsequent collapse seen in the rheology. To prepare samples for imaging, 0.2 volume % 2-hydroxy-2-methylpropiophenone (photoinitiator) was added to $\phi = 0.1$ nanoemulsion samples which were then placed between two glass microscope slides held 1 mm apart and quenched($\approx 30°C/min$) from $20°C$ on the ARG2 rheometer Peltier temperature stage to replicate the thermal environment of the rheometer samples during SAOS experiments. The samples were quenched to different temperatures and aged for various lengths of time before being cured for 5 minutes under ultraviolet (UV) light ($\approx 5mW/cm^2$) to lock in the droplet microstructure for subsequent imaging on the microscope. After curing, the samples were imaged using an Olympus IX71 microscope equipped with an Andor Clara digital camera (Andor Technology).

Images for different age times for the quench to $B_2^* = -5.60$ are shown in Fig. S5(b). Individual nanoemulsion droplets which are around 50nm in diameter are unresolvable using light microscopy, so at early times before larger resolvable clusters and phase separated structures have formed, nothing is observed in the microscopy. At 10 minutes, when the elastic modulus reaches a peak, still no structure is resolvable in the microscopy which suggests that the network strands are tenuous with characteristic length scales below the resolution limit of the microscope. However, as the gel collapses tiny speckles start to appear. These speckles continue to grow in number and size as phase separation proceeds unarrested.

Similar rheological behavior and evolution of structure are observed at other quench depths as shown in Fig. S6(a) and (b). The observed gelation time is quench depth dependant with gelation shifting to longer times as the quench depth is decreased. Consistent with the rheology, the appearance of resolvable structure in the microscopy is also quench depth dependent with structures appearing at longer times as the quench depth is decreased. At long times, macroscopic phase separation and droplet coalescence are observed as indicated with the yellow circles in Fig. S6(b), consistent with our hypothesis that coarsening proceeds unarrested after gel collapse. In addition to droplet coalescence, droplet sedimentation was observed. While the continuous phase and dispersed phase are very close in density allowing individual nanoemulsion droplets to remain well dispersed, when these droplets aggregate into larger clusters or coalesce into larger droplets, the small difference in density between phases causes droplets to sediment if a strong elastic network of droplets hasn't formed. This sedimentation was experimentally observed as a small pool of oil at the top of the samples at the end of the rheological test.

The experimentally observed gel collapse represents an additional region in the experimental phase diagram, labeled V_A in Fig. S6(c), that is not observed in the simulations. The transition from the gel collapse region to the region where persistent gels form is demarcated with a dashed blue line in Fig. S6(c). Similar gel collapse behavior has also been observed in poly-methylmethacrylate (PMMA) colloid systems with depletion interactions studied by Harich and Poon et al. (4).

1.6 Experimental Gelation Line, Equilibrium Phase Boundary and Percolation Line. To verify whether the onset of gelation for the experimental system corresponds with the equilibrium phase boundary and the percolation line, the experimental gel line was compared with these transitions. The equilibrium phase boundary was determined for the experimental SW potential shown in Fig. 2(b) using previously published literature by del Río (5), Vega (6), Elliot and Hu (7) and is shown in Fig. S6(c) with closed blue triangles. Similarly to the 2Y results discussed in the main text, the experimental gel line (solid blue line with square symbols) transitions from being roughly horizontal at intermediate ϕ to being positively sloped at high ϕ when it intersects the equilibrium phase boundary at the T_g^{sp} point.

To contextualize the low ϕ downturn in the gel line, the pair connectedness percolation line was also determined for the experimental system using previously published literature by Netemeyer et al. (8) and is shown in Fig. S6(c) with a solid green line with open green square symbols. The percolation line from Netemeyer ends at the phase boundary, but has been extrapolated into the phase instability region as shown with the green dashed line in Fig. S6(c). Interestingly, the experimental gel line does not turn down immediately adjacent to the pair percolation line from Netemeyer, but instead, it turns down at a higher ϕ (as indicated with an arrow in Fig. S6(c)). This is different from the 2Y results discussed in the main text where the gel line turned down immediately adjacent to the isostatic percolation line. One reason for this difference comes from the definition of percolation that is being used for the experimental and 2Y systems. While the isostatic line was determined for the 2Y, isostatic percolation lines for the SW potential have not been determined in the literature, so here the pair connectedness percolation line from Netemeyer was used. Pair connectedness percolation is a less stringent condition for percolation than isostatic percolation and only describes the static connectivity properties of the system, i.e. is there a space spanning network

of particles, it does not take into account the local rigidity of clusters in that network. Hence, pair percolation lines occur at lower volume fractions than isostatic percolation lines. Because of this, the pair percolation line can only be used to establish a lower bound on where gels occur in the experimental phase diagram.

Interestingly, while gel collapse is observed adjacent to the percolation line in the experimental system, no gel collapse is observed in the simulations, and gels persist everywhere to the right of the percolation line for quenches below the T_g^{sp} point. Harich and Poon et al. (4) had also observed a difference between the proximity of the percolation line to the region where persistent gels form in simulation and experiments, and suggested that gravity accounts for this difference. In finite gravity, the region where persistent gels form "should lie to the right of the percolation boundary" but in the simulations where there is zero gravity present, gels should be observed "immediately to the right of the percolation line inside the spinodal" (4). This reasoning is in agreement with the differences that we observed between the experimental system and the simulations.

1.7 Quantifying the Bridging Polymer Concentration Distribution Using MRI. To ensure that droplet interactions arising from the PEGDA bridging polymer were uniform throughout the sample during phase separation, it was necessary to determine whether the polymer concentration in the droplet dense and droplet dilute phases were equivalent. If the polymer preferentially partitions into either phase, this will impact the slope of the equilibrium tie-lines as droplet interactions will be stronger in the phase containing a higher concentration of polymer. To check whether or not this was the case, the polymer concentrations in the droplet dense and droplet dilute phases were quantified using the experimental method discussed below. A $\phi = 0.33$ nanoemulsion sample was placed in a centrifuge tube and quenched from $25°C$ ($B_2^* = -0.80$) to $50°C$ ($B_2^* = -4.75$) in an oven, then aged for 30 minutes to allow gelation to occur. Once the gel formed, the sample was immediately centrifuged at 9000 x g for 20 minutes to separate the dense and dilute phases. The centrifuged sample was then imaged using a Bruker 300MHz (7 T) super-wide-bore (SWB) Magnetic Resonance Imaging (MRI) spectrometer. By tuning to the frequency of the ethylene glycol proton shift on the PEGDA, the distribution of PEGDA in the dense and dilute phases of the nanoemulsion could be determined. This process is shown in Fig. S18. In (a), the gelled nanoemulsion sample is shown before centrifugation. In (b), the sample has been centrifuged and a clear interface between the droplet dense region on top and droplet dilute region on bottom is visible. In (c), a vertical cross section of the centrifuge tube was imaged using MRI. In (d), an intensity profile is shown for a vertical slice through the middle of the centrifuge tube which has been indicated in (c) with a dashed line. The color intensity is uniform throughout both phases indicating that the concentration of PEGDA is also uniform. Interestingly, while the concentration of PEGDA is uniform in both the droplet dense and dilute phases, there is a greater intensity of PEGDA at the interface as can be seen in (d). This is likely because the density of PEGDA is intermediate to that of oil (which makes up most of the droplet rich phase and has the lowest density), and water (which makes up most of the droplet dilute phase and has the highest density). So, the higher concentration of PEGDA at the interface is an artifact of the centrifugation. Nevertheless, this experiment confirms that the colloid interactions from polymer bridging remain constant at fixed temperature during phase separation as the PEGDA is uniform in the droplet dense and droplet dilute phases. This also confirms that the orientation of the equilibrium tie-lines in the experimental phase diagram are horizontal.

2. Simulation Systems

2.1 Two-Yukawa and Morse Potential Parameters. Two interparticle potential models, $V_{ij}(r_{ij}|T)$ were used in this work to describe the interactions between particles in simulations, the Two-Yukawa (2Y) and Morse potential. These potentials are defined below:

Two-Yukawa:

$$\frac{V_{ij}(r_{ij}|T)}{k_bT} = \begin{cases} \infty, & r_{ij} \le a_i + a_j \\ -K_1 \frac{e^{-Z_1[r_{ij}-(a_i+a_j)]}}{r_{ij}} + K_2 \frac{e^{-Z_2[r_{ij}-(a_i+a_j)]}}{r_{ij}}, & r_{ij} > a_i + a_j \end{cases} \quad [2]$$

where r_{ij} is the center-to-center distance between particles i and j with radii a_i and a_j, T is temperature, and k is Boltzmann's constant. The 2Y has four adjustable parameters: $K_1(T)$ and $K_2(T)$ characterize the magnitude of the attractive and repulsive interactions respectively, while $Z_1(T)$ and $Z_2(T)$ characterize the range of the attractive and repulsive interaction respectively. Part of the utility of the 2Y potential comes from its ability to capture both attractive and repulsive interactions of varying range allowing it to describe a diverse range of systems.

In the current work, K and Z parameters were chosen to produce a 2Y potential with a short range repulsion near contact followed by a longer range attraction. For the 2Y potential shown in Fig. 1(d), $K_1 = 11.03$, $K_2 = 11.05$, $Z_1 = 12.56$, and $Z_2 = 24.6$. To modify the potential attraction strength the K_1 parameter was changed while holding the other parameters (K_2, Z_1, Z_2) fixed. By increasing the magnitude of K_1, phase separation and gelation can be induced.

Morse:

$$\frac{V_{ij}(r_{ij}|T)}{k_bT} = -\frac{V_0}{k_bT}[2e^{-\kappa*(r_{ij}-(a_i+a_j))} - e^{-2\kappa*(r_{ij}-(a_i+a_j))}] \quad [3]$$

where V_0/k_bT is the potential well depth and κ sets the range of the potential. Morse parameters were chosen in order to produce a shorter ranged potential relative to the 2Y and experimental SW potential to investigate whether the shape of the potential changes the observed gelation behavior. This potential was also chosen because it has been studied extensively in previous literature and because it can be used to describe systems with polymer bridging induced attractions. For the potential shown in Fig. 1(d), $V_0 = 4.4\ k_bT$ and $\kappa = 30/a$. To change the potential attraction strength, V_0 was varied within a range of $3 - 6\ k_bT$ while holding κ fixed.

2.2 Lammps Simulations. We constructed our computational model system using the LAMMPS molecular dynamics package. Our computational model system comprises a suspension of 750,000 nearly hard spheres suspended in an implicit, Newtonian solvent. The particles are neutrally buoyant with an average radius a. A polydisperse distribution of particles was used in simulations to avoid crystallization. For the Morse, five equally populated particle sizes were used giving a PDI of 0.07. For the 2Y, five equally populated particle sizes were used giving a PDI of 0.28. Fluid motion is governed by Stokes' equations due to the small Reynolds number associated to the colloidal particles, $Re = \rho U a/\eta$ where ρ is the density of particles, U is a characteristic particle velocity, and η is solvent viscosity. The volume fraction of particles $\phi = 4\pi a^3 n/3$, where n is the number density of particles, ranges from $\phi = 0.05$ to 0.65, which enables us to probe the entire phase diagram.

2.3 Simulation Rheological Characterization. SAOS was implemented in simulations with a dimensionless frequency $\alpha = \omega a^2/D = 0.5$, and strain amplitude $\gamma = 0.002$, resulting in a dimensionless Peclet number $Pe = \gamma\alpha = 0.001$. Thus, the duration of each oscillation cycle was $2\pi/\omega \sim 12.56$ Brownian times (BT). The initial configuration ($B_2^* = 1$ for both the Morse and 2Y) was instantaneously quenched to the target temperature, and simultaneously, oscillatory shear was imposed throughout the period of simulation, to reflect the experimental protocol. A total of 80 cycles were performed, resulting in a total duration of simulation of approximately 1000 BT. The elastic stresslet was measured at every instant of time, averaged over all pairs of particles, at a sampling rate of 0.004 BT. The elastic and viscous moduli were computed as an average over several cycles in order to reduce noise arising from Brownian motion. In this study, data from 10 oscillatory cycles, corresponding to a time window of 125.6 BT, are used to compute the Fourier coefficients to obtain the elastic and viscous moduli. The midpoint of each time window was assigned the computed values of the moduli. Overlapping time windows provided nearly continuous data for the moduli, beginning at the end of five cycles (which is the midpoint of first 10 cycles), approximately 62.5 BT, and ends at the end of 75 cycles (which is the midpoint of the last 10 cycles), approximately 942 BT. For high ϕ, where gelation (determined as the point of G' and G'' crossover) occurred within the first few oscillation cycles, no averaging was carried out as averaging the data would have obscured the crossover time and averaging was not needed to reduce noise. For specific data points on the phase diagram, a few additional cycles were computed if it appeared that the elastic crossover was occurring just beyond 1000 BT.

Prior studies in the literature have suggested that SAOS can enhance aging of the gels due to shear, which can result in a reduction of the measured gel time compared to the gel time arising from quiescent conditions. To ascertain whether such an effect is important for the current model system, we implemented an alternate protocol, where the gel was allowed to quiescently age for a certain duration, following which 10 cycles of oscillatory shear were performed and the moduli computed as described above. The difference between gel times obtained by the two protocols varied in magnitude, but was within a duration of a cycle. In the context of the gels examined in this study, the gel times are significantly larger than the duration of a single cycle. Therefore, the chosen protocol (concomitant aging and oscillatory shear) closely replicates the experimental protocol and is expected to be sufficiently accurate to determine the gel line.

Rheological quench data for the Morse and 2Y are shown in Fig. S1 and S2 respectively. Similarly to the experimental rheology presented in the main text, the Morse and 2Y rheology exhibits different time evolution at low, intermediate, and high ϕ. At low ϕ, deeper quench depths are required for gelation and gels form at longer age times. The resulting gels are also an order of magnitude weaker than those that form at higher ϕ. At intermediate ϕ, gels form at shallower quench depths and at shorter times. As the quench depth is decreased, gels take longer to form and the gel modulus decreases. However, at high ϕ, the gel time is quench depth independent for quenches deeper than the gel line with gels forming nearly instantaneously for the Morse and instantaneously (within the first oscillation cycle) for the 2Y.

2.4 Determination of the Simulation Percolation Line. For irreversible (chemical) gels, the gelation process and emergence of elasticity has been well described in terms of pair connectedness percolation, in which gelation occurs when an infinite space-spanning network emerges. For reversible (physical) gels, however, the static criterion of a space-spanning network is not sufficient to predict gelation as clusters break and form due to consistent rearrangement of particles. To address this issue, Tsurusawa et al. (9) sought to find a direct correlation between percolation of particles and emergence of elasticity in reversible colloidal gels. He found that the percolation of isostatic structures, the emergence of a network of particles with at least six bonds, portends the emergence of mechanical stability or rigidity.

Following the approach of Tsurusawa, in this work we detect the emergence of the percolation of isostatic particles to construct a isostatic percolation line for the 2Y potential (green line with squares in Fig. S8(a) and Fig. 4 in the main text). To save computational resources, the isostatic percolation line was not determined for the Morse simulations; however, the same qualitative behavior is expected for percolation in the Morse system as illustrated with a sketch of the percolation line on the Morse phase diagram in Fig. S10(a) (dashed green curve).

To determine the isostatic percolation line for the 2Y, we detect the combinations of ϕ and B_2^* that correspond with the emergence of the percolation of isostatic particles (particles forming at least six contacts with neighboring particles, $N_C \geq 6$). Formation of six neighbors satisfies the local Maxwell criterion for isostaticity that limits all internal degrees of freedom of a particle (10). For each point in phase space tested (ϕ, B_2^*), instantaneous simulation quenches were performed from $B_2^* = -1.0$ to the final quench depth followed by aging for 1000 BT. During the last 400 BT of aging, 10 simulation snapshots, equally spaced over that interval were obtained. Isostaticity was then determined for each snapshot by first computing N_C for each particle in the snapshot. Next, particles with at least six contacts were marked as "isostatic" and then it was determined whether or not a space spanning network of isostatic particles was present. Particle snapshots used in this analysis are shown for three ϕ in Fig. S12 with the location where renderings were taken indicated on the phase diagram in Fig. 4 with a green

box. The top row of images in Fig. S12 show all simulated particles with particles colored according to N_C. The bottom row of images show particle renderings after non-isostatic particles ($N_C < 6$) and small clusters have been removed with particles colored according to which cluster they belong to. For $\phi = 0.05$, small discrete clusters are observed. At $\phi = 0.12$, multiple larger clusters (containing at least 1% of all isostatic particles) exist and at least one of these clusters has formed a percolated network. At higher particle density, $\phi = 0.15$, a majority of the isostatic particles (more than 99% of all isostatic particles) are connected in one dominant cluster that percolates the system. In general, we observe that systems with percolating isostatic clusters can be categorized into two typical cases: (1) where there exists multiple large clusters of isostatic particles and at least one of them percolates; (2) where there exists exactly one large cluster of percolating isostatic particles. The later case occurs for more crowded (high ϕ) systems and V(r) with more negative B2 (more attractive). Here we use case (2) as the criterion for isostatic percolation. See Fig. S13 for additional simulation snapshots used in this analysis.

2.5 Determination of the Simulation Phase Boundaries. Three approaches were used to determine the location of the phase envelopes for the simulation systems: (1) the coexistence simulation method of Statt and Panagiotopoulous (11) (2) the analysis of simulation snapshots at various ϕ and B_2^* to visually determine the boundary of phase separation, and (3) the analysis of particle contact distributions, $P(N_C)$, at various ϕ and B_2^*. Each approach is discussed in detail below.

2.5.1 Coexistence Simulations For the 2Y and Morse potentials, the equilibrium phase boundaries (black and red dashed line with triangle symbols in Fig. S8 and S10 respectively) were determined by adapting the coexistence simulation method of Statt and Panagiotopoulos (11). Briefly, to determine the phase envelope using this method, an elongated simulation box oriented vertically was initialized with a dense region ($\phi_L = 0.6$) sandwiched between two dilute regions that initially contain no particles ($\phi_V = 0.0$). The total volume fraction of particles in the simulation box was $\phi = 0.257$. With the system initialized, the system was instantaneously quenched and then aged until an equilibrium between the dense and dilute phases was reached, after which the volume fraction of each phase was calculated to determine the location of the phase boundary for that quench depth. This process was repeated for other quench depths to determine the entire phase boundary. An example of this analysis is presented in Fig. S7(b) for the 2Y system and shows simulation snapshots of the simulation box for a quench to $B_2^* = -2.6$ at several different BT. At early BT, particles are quickly ejected from the dense phase and the dense phase expands in volume as can been seen from the shift in the location of the interfaces. This indicates that the initial dense phase volume fraction is to the right of the high density branch of the binodal and thus has to reduce its density to reach equilibrium. At later BT, the location of the interfaces stop changing indicating that equilibrium has been reached. During these simulations, the local volume fraction was calculated as a function of height, z, within the simulation box as shown in Fig. S7(a). The volume fraction of the dense phase rapidly decreases at early BT and then equilibrates thus corroborating the findings from visual inspection of snapshots as discussed above. Once equilibrium was reached, the volume fractions of the dense and dilute phases were calculated by averaging the local volume fractions within each phase. These calculations were done sufficiently far from the interfaces to ensure accurate determination of the phase boundary. From these coexistence densities, the location of the critical point was determined through fitting the data using the procedure of Statt and Panagiotopoulos (11).

2.5.2 Simulation Snapshots Simulation snapshots were strategically taken at two different locations in the phase diagram to identify the quench depth of the critical point and to determine where the gel line intersects the phase boundary. Simulation particle snapshots were collected at various ϕ and B_2^* under quenching from an initially uniform density and visually analysed to determine where the onset of phase separation occurred. The particle renderings are shown in Fig. S8(b) and S10(b) for the 2Y and Morse respectively. The location in phase space where particle snapshots were collected are indicated with star symbols in the phase diagrams in Fig. S8(a) and S10(a) for the 2Y and Morse respectively. Particles in renderings are colored according to contact number as previously described, with blue particles having many contacts, white particles having a few contacts and red particles freely diffusing. For shallow quenches, particles are dispersed with only a few particles having multiple contacts (mostly red colored particles with a few white particles). As the quench depth increases, particles start to phase separate to form a dense phase. This is seen in the renderings as the formation of particle strands with interior particles having large particle contact numbers (more blue particles). The location where the transition from fluid to the two-phase region occurs is indicated with a dashed red line between particle renderings.

To determine the location of the dense branch of the phase boundary near the gel line, ϕ was systematically increased at fixed B_2^* and particle renderings (shown in Fig. S8(c) and S10(c)) were visually analysed to determine the ϕ where phase separation was no longer occurring. Within the phase envelope, the thermodynamic driving forces result in the formation of a colloid-rich phase and colloid-poor solvent regions. Since the tie-line is horizontal, one can sample the tie-line by increasing the overall volume fraction of the colloids while keeping the quench depth fixed. Based on the lever rule, one expects that as the overall volume fraction increases, the overall volume of the solvent pores decreases and ultimately vanishes at the binodal. We use this principle to estimate the dense-phase binodal at the quench depth of the horizontal gel line. Visual inspection of the snapshots shows a clear reduction in the overall volume of solvent pores as volume fraction is increased. At high ϕ just inside the phase boundary, small voids surrounded by a dense sea of particles grow in time, which is characteristic of phase separation. However, as ϕ is increased and the phase boundary is approached, these voids get smaller and smaller. Outside the phase boundary, neither structural coarsening nor voids are observed, suggesting glass like behavior. The location of the transition from the two-phase region to the attractive glass region, where no solvent pores are observed, is indicated with a blue dashed line.

Both the quench depth of the critical point and the ϕ location of T_g^{sp} determined from analysis of particle snapshots (as marked by the red and blue dashed lines respectively) are consistent with the phase boundary determined from the coexistence

simulations, thus confirming that the location of the phase boundary has been correctly identified.

2.5.3 Particle Contact Number Distributions To determine the quench depth of the critical point using particle contact number probability distributions, $P(N_C)$, the time evolution of $P(N_C)$ was studied over a range of quench depths for $\phi = 0.2$ to determine where bimodal distributions of particle contacts occurred. The formation of bimodal distributions in $P(N_C)$ suggest the formation of an equilibrium dense and dilute phase. $P(N_C)$ plots are shown in Fig. S9 and S11 for the 2Y and Morse respectively. Four types of $P(N_C)$ time evolution were observed at the quench depths tested. 1) At shallow quenches (panel (a)), the $P(N_C)$ distributions quickly converge within 40 BT to the final distribution. The final mean contact number is also low ($N_C < 4$) indicating the system contains mostly freely diffusing particles and small clusters, but not large networks of particles. This behavior is indicative of a quench in the homogeneous fluid region of the phase diagram. 2) As the quench depth is increased (panel (b)), distributions no longer converge quickly to an equilibrium distribution. Instead, the peak of the distribution broadens and shifts downwards and to the right, continuing to evolve even after 1000 BT. This behavior is indicative of a quench near the critical point as the driving force for phase separation is smallest here and evolution of structure towards equilibrium macroscopic phase separation is slow. The quench depth where this behavior was observed is consistent with the quench depth of the critical point determined in the coexistence simulations and from analysis of simulation snapshots. 3) As the quench depth is further increased (panel(c)), distributions still do not converge to an equilibrium distribution within 1000 BT. However, unlike panel (b), at this quench depth the driving force for phase separation is larger and a bimodal distribution starts to form at long times indicating the presence of a dense and dilute particle phase. Also, $P(N_C = 0)$ grows in time indicating the creation of freely diffusing particles in the dilute phase while the peak location at $6 < N_C < 8$ suggests liquid like regions. This behavior is indicative of a quench to the two-phase coexistence region but above the gelation line. 4) In panel (d), the peak evolves rapidly down and to the right ($N_C \geq 8$) while freely diffusing particles are depleted indicating the formation of a gel as the bond lifetime is long and most particles are densely packed with each particle having many contacts.

3. Dependence of the State Diagram on Interparticle Potential

While the three model systems all produce similar qualitative behavior with respect to the location of the gel line relative to the equilibrium phase boundary (main text Fig. 5), there exist quantitative differences between the locations of the gel lines in phase space that depend on the shape and range of the potential. For example, both the location of T_g^{sp} and its associated volume fraction that set the transition between the intermediate and high ϕ regions of the gel line depend on the range of the potential. This can be simply explained by the expected dependence of the location of the glass line on the range of the potential. Specifically, an attractive glass emerges because of "crowding" of interparticle bonds, which arrest the dynamics of bond rearrangement (12–14). Consequently, as the range of attraction is increased, then a lower ϕ is needed in order for crowding of bonds to occur. Thus, the location of the line demarking the glass transition is expected to shift to lower ϕ as the range of attraction increases, and this is what is observed for the segment of the gel line for values of ϕ to the right of the binodal in Fig. 5(a). We note that a similar phenomenology explains the dependence of the location of the dense branch of the vapor-liquid binodal on the attraction range. Bergenholtz et al. (13) and Dawson et al. (14) observed this same behavior, where the ϕ location of the glass line at low attraction strength (determined from mode-coupling theory (MCT) for attractive Yukawa and SW potentials) increased with decreasing range of potential. While these predictions of the glass line by MCT glass give insight into how the high ϕ branch of the gel line depends on shape of the interaction potential, MCT theory alone does not accurately capture gelation behavior at low and intermediate ϕ because MCT cannot correctly describe phase separation (15). To address the shortcomings of MCT, new theoretical frameworks are being developed to account for this low ϕ behavior(16).

In addition to its location, the slope of the glass line is also potential-dependent. However, in the present work, this could be due to a combination of several factors, including differences in polydispersity between the models, the range of the potential, and the nature of the repulsion near contact. The individual influence of each of these factors on the slope of the glass line outside the phase boundary is left for ongoing studies.

While the range of the interaction potential accounts for the shift in the glass line at high ϕ, the vertical location of the gel line at intermediate ϕ (set by T_g^{sp}) also changes monotonically with the attraction range when gel lines are compared in K_bT/V_0 (Fig. 5(a)) space but not monotonically when compared in B_2^* space ((Fig. 5(b)). Similar behavior is seen with the equilibrium phase boundary, which shifts monotonically upward with increasing range in k_bT/V_0 space, but overlap each other in B_2^* space. This is consistent with what is observed for SW potential phase boundaries when the range is increased (Fig. S17(a) and (b)).

At low ϕ, the gel lines are steeply sloped and follow the percolation line. Thus, the change in gel line with attraction range can be simply explained by the range-dependence of the percolation threshold. For example, Netemeyer et al. (8) examined the location of pair connectedness percolation transition for SW potentials of varying range and found that as attraction range (well width) increases, the percolation transition shifts to lower ϕ, as expected due to the longer "bond length" for longer-range attractions, such that longer-range attractions percolate to lower densities. While to our knowledge no analogous detailed studies exist for the isostatic percolation threshold, we expect a similar trend for isostatic percolation. While the experimental and simulation data presented here are sparse at low ϕ, it appears that the location of the low ϕ branch of the gel shifts to lower ϕ with increasing attraction range, consistent with this expectation.

As discussed in the main text, neither B_2^* nor k_bT/V_0 collapse the equilibrium nor the nonequilibrium gel lines. Instead, we discovered that rescaling ϕ and B_2^* by their respective critical point values, ϕ_c and $B_{2,c}^*$, respectively, approximately collapses the phase envelopes and gel lines. Despite the fact that the three distinct regions of the gel line have different associated

dominant physics, it can be argued that each of these dominant mechanisms (percolation, phase separation, glassy arrest) are expected to qualitatively scale similarly with the range of the interaction potential, and so can be tracked with the location of the critical point. For example, both the percolation threshold and the location of T_g^{sp} relative to the equilibrium phase boundary are expected to shift to lower ϕ as the range of the attractive potential increases for the reasons discussed above. Because ϕ_c also shifts to lower values with increasing attraction range (Fig. 5(a)), ϕ_c appears to provide an adequate proxy for the location of the percolation and attractive glass lines.

4. Insensitivity of the Location of the Gel Line to Applied SAOS Frequency

For the experimental and simulation systems, the gel line was determined by performing an extrapolation of quench depth (B_2^*) vs inverse gel time (τ_{gel}^{-1}) data (Fig. S3) collected from SAOS rheological tests performed at a fixed oscillation frequency. To investigate whether the location of the gel line determined using this method was sensitive to the applied SAOS oscillation frequency, frequency sweeps were performed for the 2Y system (Fig. S4(a)) at a fixed strain amplitude of $\gamma = 0.002$. In these tests, quenches were performed at $\phi = 0.3$ from $B_2^* = 1.0$ to three quench depths, one quench to just below the percolation line ($B_2^* = -1.1$), another quench to the location of the gel line determined from initial SAOS measurements ($B_2^* = -3.6$), and a deep quench below the gel line ($B_2^* = -12.0$). Following the quench, the systems were aged for 1000 BT after which a frequency sweep was performed. For quenches just deeper then the percolation line, the frequency response was dominantly viscous over most frequencies with G' and G'' having a slope of 2 and 1 respectively (terminal viscoelastic behavior). Only at really high frequencies (100 $\frac{1}{(a^2/D)}$) was there a dominantly elastic response. For quenches to the gelation line, the frequency response was dominantly viscous below 1 $\frac{1}{(a^2/D)}$ but dominantly elastic above this frequency with G' and G'' having a slope of about 1/2, consistent with the Winter and Chambon criteria for the gelation transition (17, 18). For the deep quench, G' was greater than G'' over all frequencies with both being less sensitive to frequency with a slope around 1/6. To confirm the simulation results, similar tests were also performed in experiments for $\phi = 0.33$ samples, where samples were quenched to various depths and aged for two hours after which a frequency sweep was performed (Fig. S4(b)) at a strain amplitude of $\gamma = 0.1\%$. Like the simulations, the experiments showed distinctly different frequency dependant behavior above, at, and below the gelation point ($B_2^* = -2.62$). These tests suggest that the location of the gel line is not frequency dependent and cannot be made to coincide with the isostatic percolation line at low attraction strengths even if higher frequencies are used during SAOS, because even at high frequencies a dominant elastic response isn't observed near the percolation line.

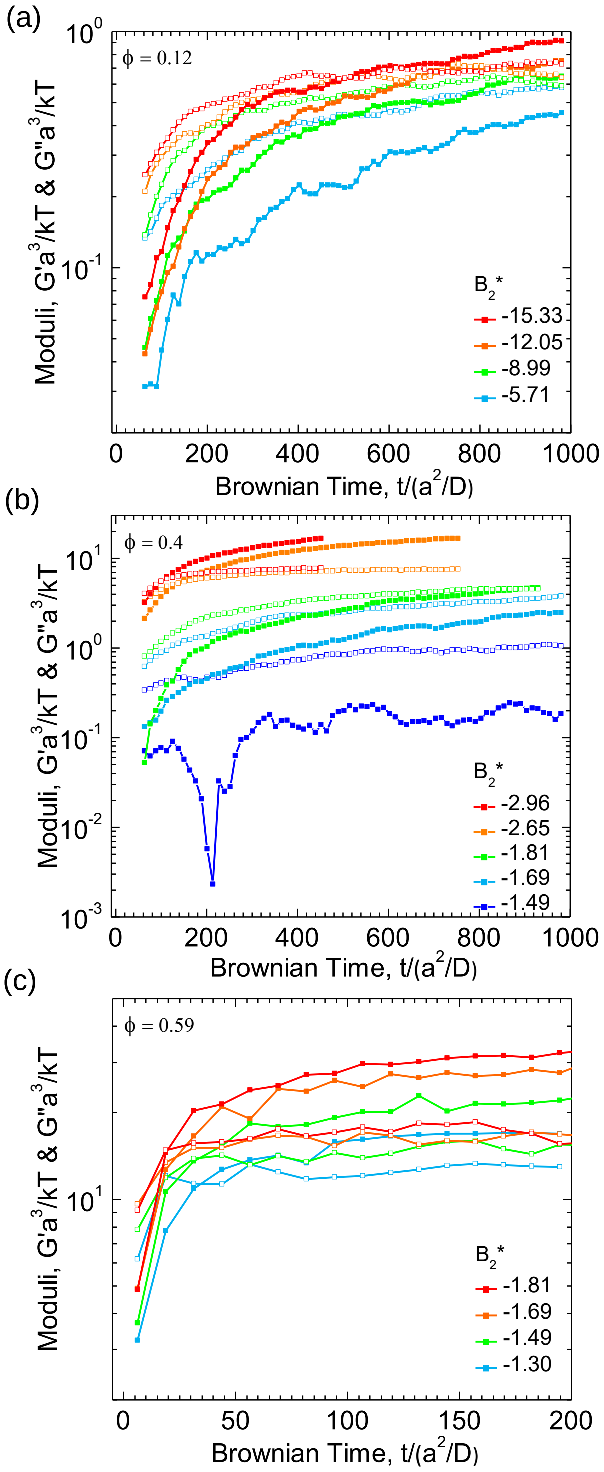

Fig. S1. Morse linear viscoelastic moduli, G' (closed symbols) and G'' (open symbols), for (a) $\phi = 0.12$, (b) $\phi = 0.4$, and (c) $\phi = 0.59$ at various quench depths

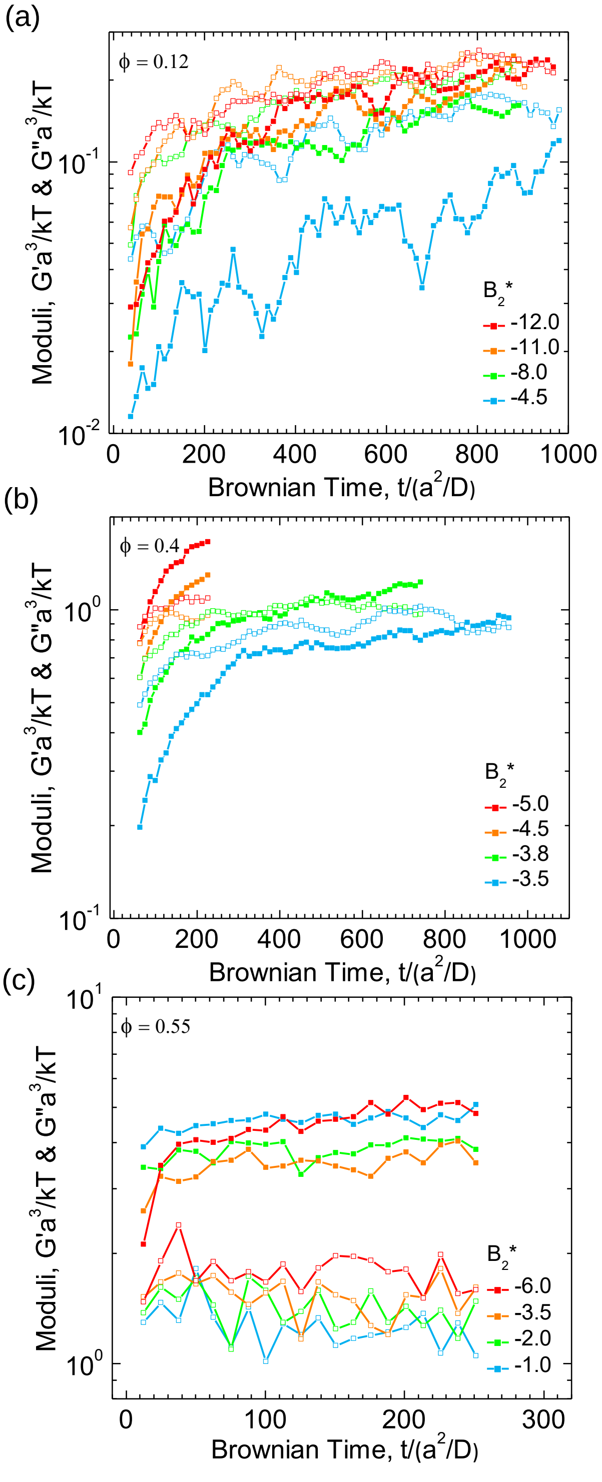

Fig. S2. 2Y linear viscoelastic moduli, G' (closed symbols) and G'' (open symbols), for (a) $\phi = 0.12$, (b) $\phi = 0.4$, and (c) $\phi = 0.55$ at various quench depths

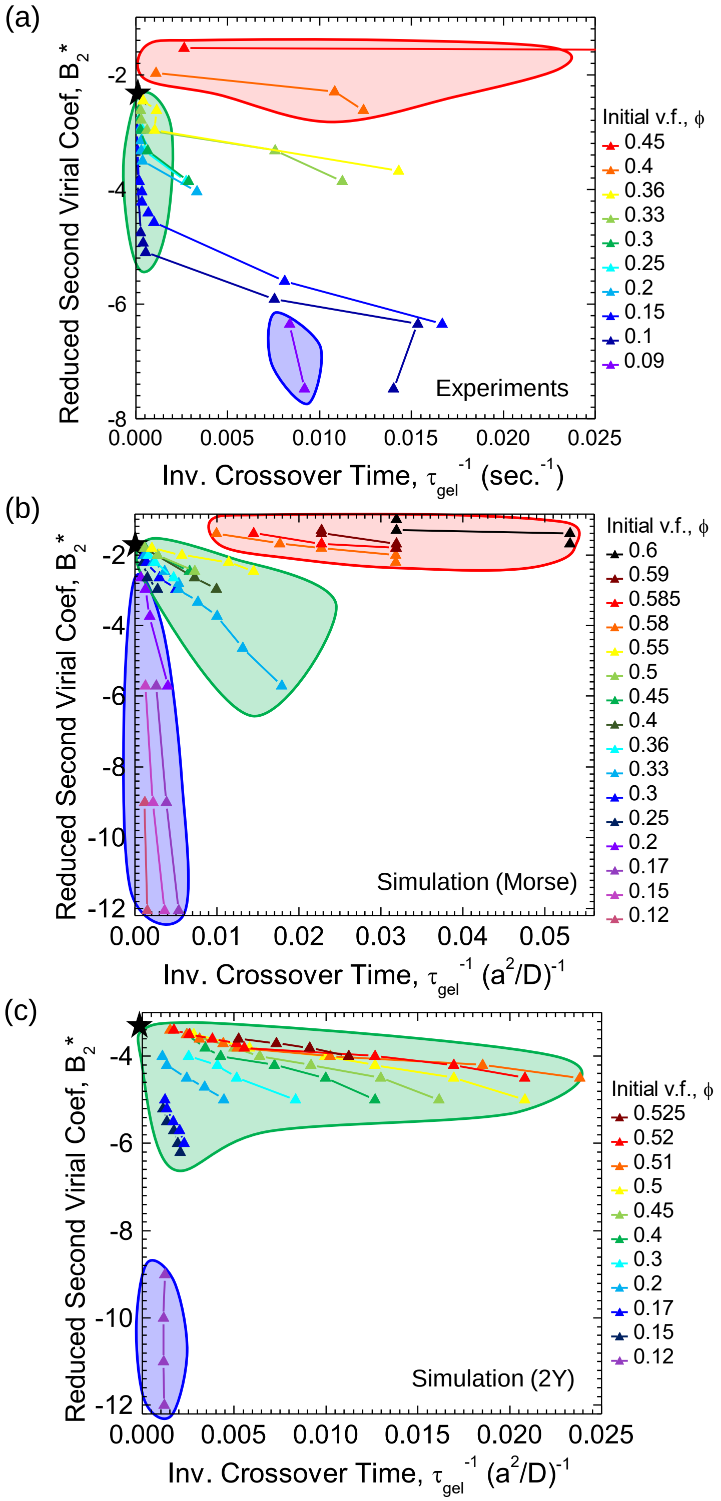

Fig. S3. B_2^* vs. the inverse of the moduli crossover time, τ_{gel}^{-1}, extracted for each quench depth and ϕ rheological data was collected at for (a) the experimental system, (b) the Morse simulations and, (c) the 2Y simulations. Three distinct regions are observed and are color-coded: a low ϕ region (blue) where curves are steeply negatively sloped, an intermediate ϕ region (green) where curves are gently sloped and approach a single point on the y-axis (marked with a black star), and a high ϕ region (red) where curves are nearly horizontal. Data from these plots were used to construct the gelation lines for each system (see main text for more details).

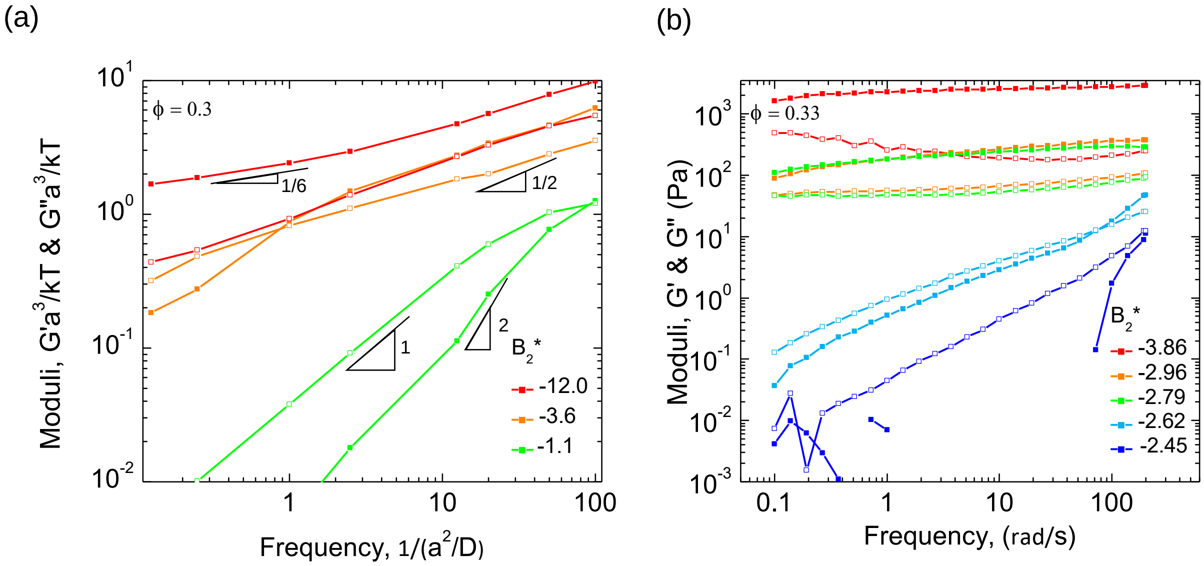

Fig. S4. Linear viscoelastic moduli, G' (closed symbols) and G'' (open symbols), from frequency sweep measurements performed at various quench depths for (a) the 2Y, and (b) the experiments to determine the sensitivity of the gelation threshold to applied frequency. Within (a), the slopes of G' and G'' are indicated.

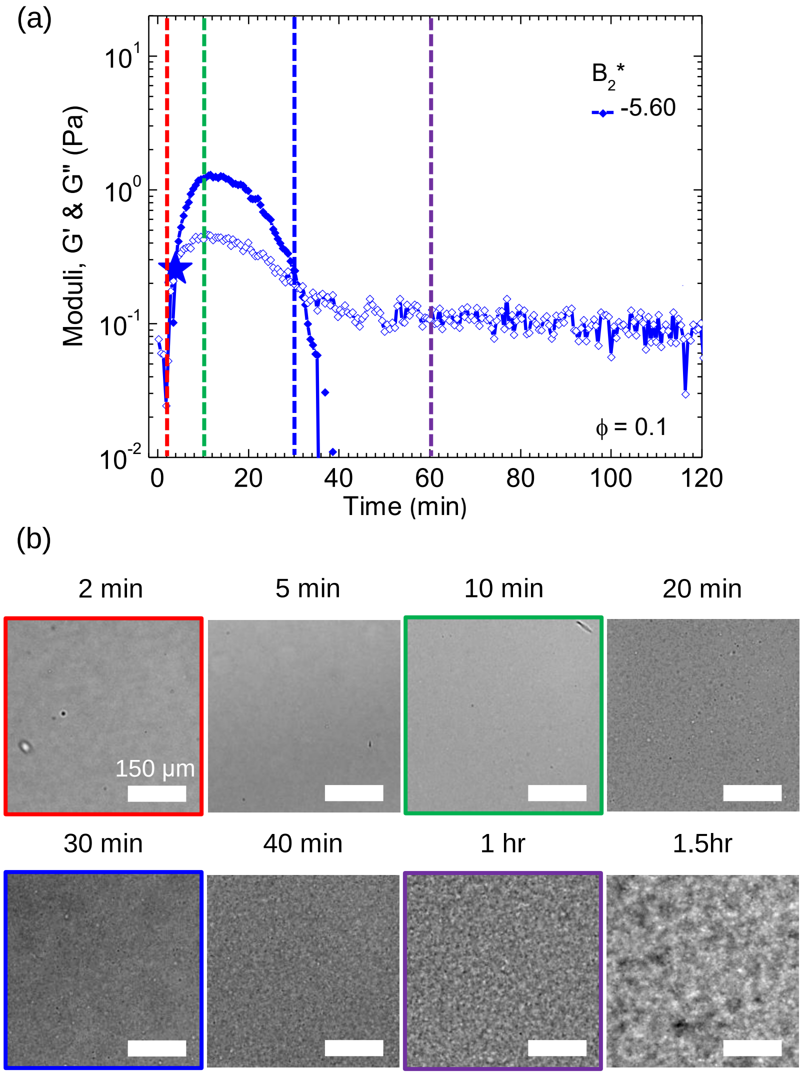

Fig. S5. (a) Experimental linear viscoelastic moduli, G' (closed symbols) and G'' (open symbols), for a $\phi = 0.1$ sample quenched to $B_2^* = -5.60$. (b) Inverted light microscopy images of $\phi = 0.1$ samples at different age times after undergoing the same thermal quench as the rheometer sample from (a).

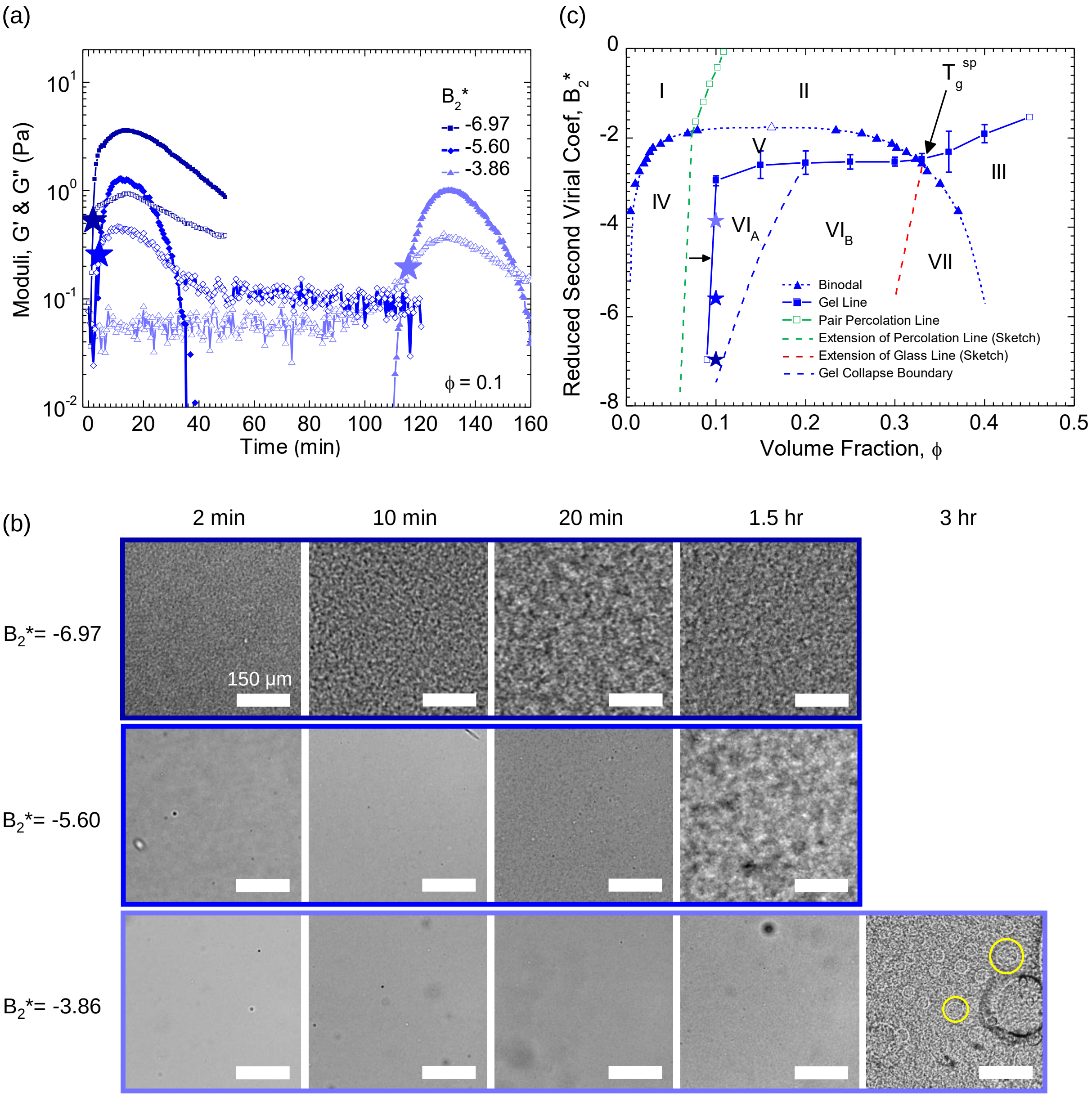

Fig. S6. (a) Experimental linear viscoelastic moduli, G' (closed symbols) and G'' (open symbols), for $\phi = 0.1$ samples quenched to various depths within the gel collapse region (b) Inverted light microscopy images of $\phi = 0.1$ samples at different age times after undergoing the same thermal quenches as the rheometer samples from (a). (c) Experimental nanoemulsion phase diagram. Solid blue triangles indicate the phase envelope reproduced from SW fluid simulation work by Vega (6), Elliot and Hu (7), and del Río (5) for a SW potential with range, $\lambda = 1.5$. The simulation data is fit using a Wegner expansion (19) (fit shown as dashed blue line). The open blue triangle indicates the critical point determined by Orkoulas and Panagiotopoulos (20). The solid green line with open green squares is the SW pair connectedness percolation line reproduced from Netemeyer (8). The percolation line from Netemeyer ends at the phase boundary, but has been extrapolated into the phase instability region (green dashed line). The experimental gel line is indicated with the solid blue line with squares. The dashed blue line demarcates the boundary between the gel collapse region (V_A) and the intransient gel region (V_B). The dashed red line is a sketch of the attractive glass line extending into the phase instability region. Stars indicate where rheology and microscopy from (a) and (b) were taken.

(a)

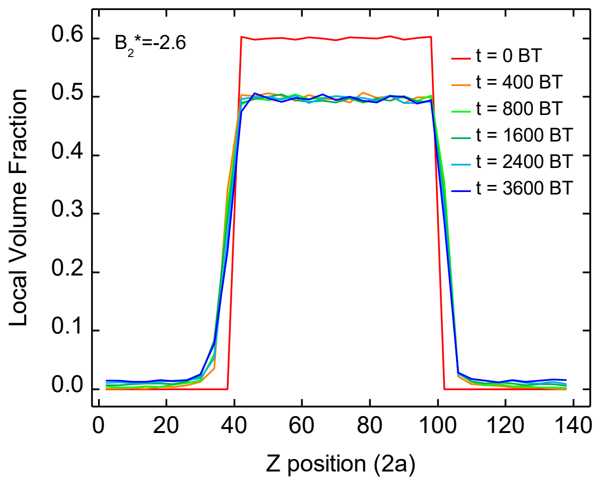

(b)

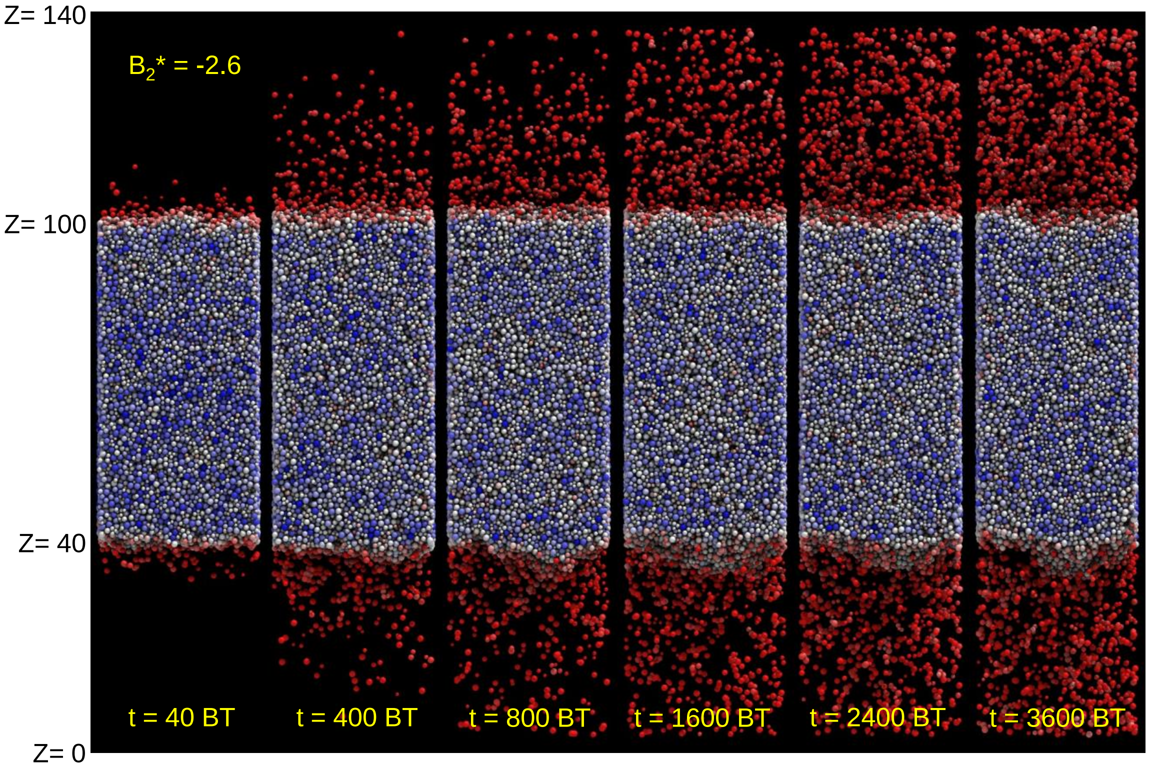

Fig. S7. (a) Local volume fraction as a function of height, Z, within the coexistance simulation box for the 2Y potential after a quench to $B_2^* = -2.6$. Local volume fraction profiles are shown at various age times after the quench. To determine the coexistence densities from this data, the local volume fractions in each phase were averaged after the system had reached equilibrium. Averaging was done sufficiently far from the interface to ensure the coexistence points were accurately determined. This analysis was repeated for a range of quench depths to determine the complete coexistence boundary. (b) Snapshots of the simulation box used to determine the local volume fraction profiles in (a).

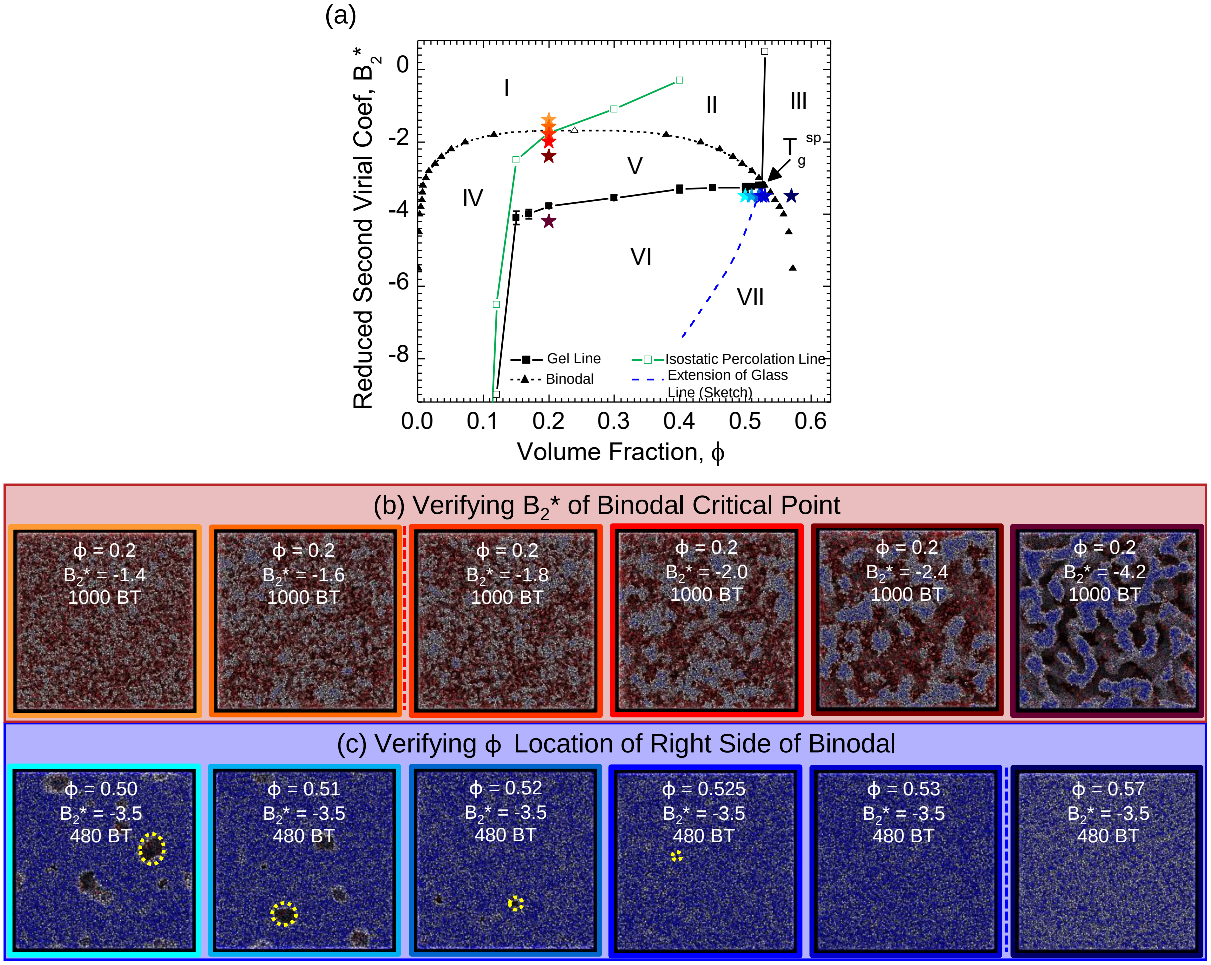

Fig. S8. Confirming the location of the 2Y phase boundary by visualization of particle snapshots near the critical point and T_g^{sp}. (a) 2Y phase diagram with binodal points (solid black triangles) determined from coexistence simulations. The critical point (open black triangle) was determined by fitting the coexistence points (with fit shown as the dashed black line) following the procedure of Statt (11). The solid black line with squares is the 2Y gel line determined from rheological quench data. The green line with squares is the percolation line determined using the method of Tsurusawa (9), and the dashed blue line is a sketch of the glass line extending into the phase instability region. Star symbols indicate where snapshots in (b) and (c) were taken. (b) Particle snapshots taken near the critical point determined from the coexistence simulations to confirm its location. The vertical dashed red line indicates the transition from the homogeneous fluid to the phase instability region. (c) Particle snapshots taken near T_g^{sp} to confirm the location of the dense branch of the phase boundary determined from coexistence simulations. Yellow circles indicate the location of particle free voids. The vertical dashed blue line indicates the transition from the phase instability to the homogeneous attractive glass region.

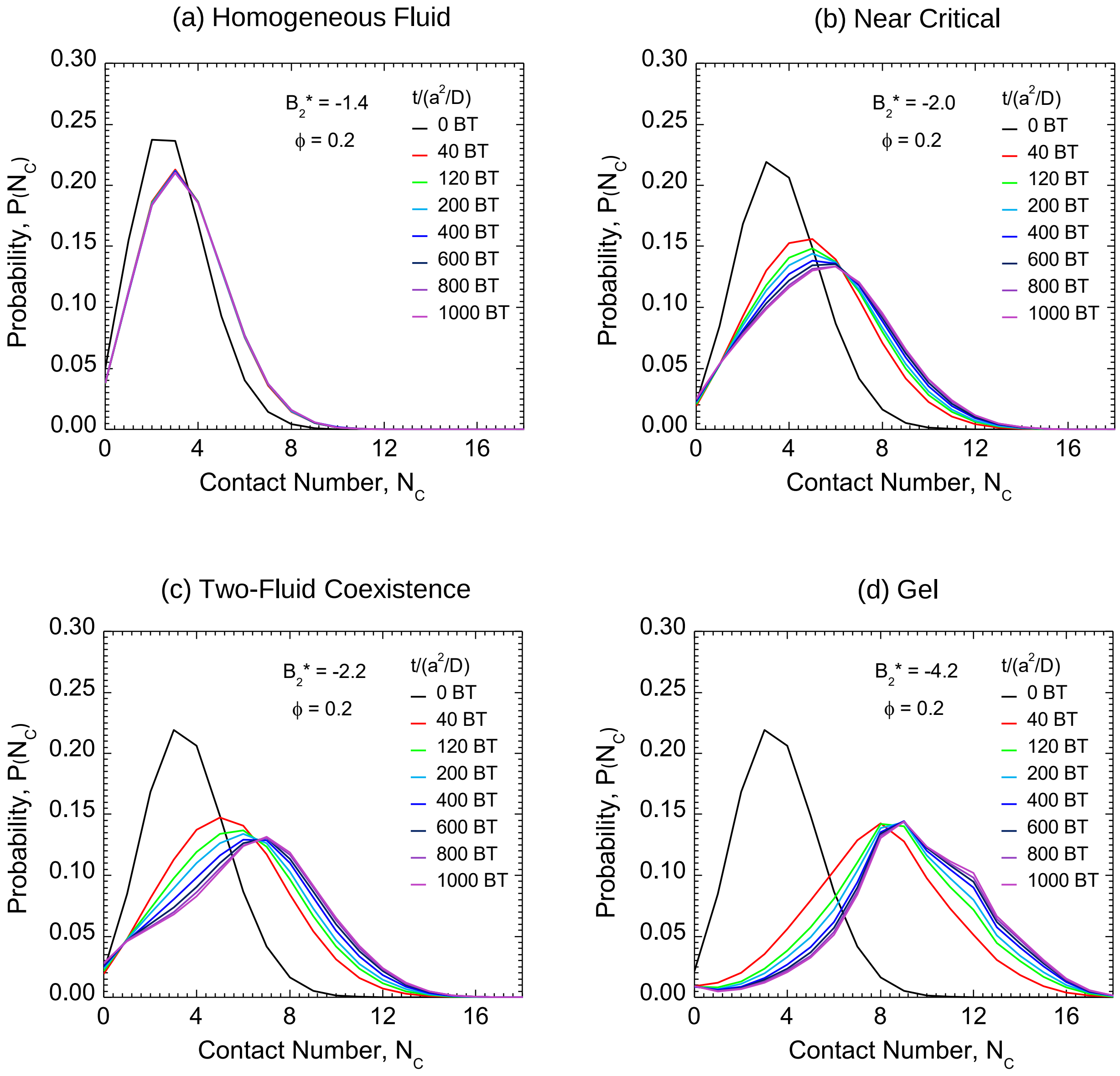

Fig. S9. Time evolution of particle contact number distributions for the 2Y for 4 different quench depths at $\phi = 0.2$: (a) a quench in the homogeneous fluid phase (b) a quench to near the critical point (c) a quench to the two-fluid coexistence region between the phase boundary and the gelation line and (d) a quench below the gelation line.

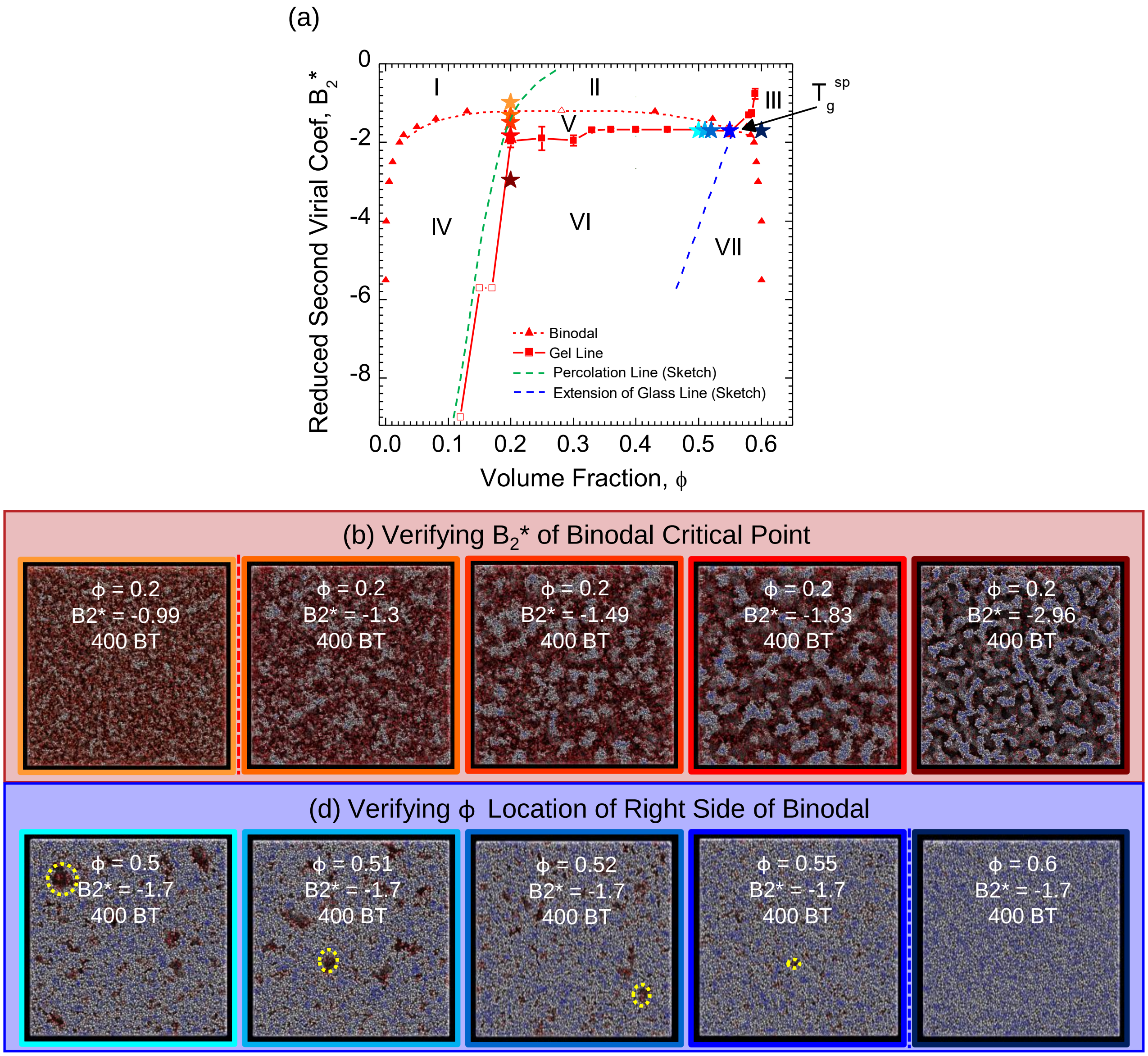

Fig. S10. Confirming the location of the Morse phase boundary by visualization of particle snapshots near the critical point and T_g^{sp}. (a) Morse phase diagram with binodal points (solid red triangles) determined from coexistence simulations. The critical point (open red triangle) was determined by fitting the coexistence points (with fit shown as the dashed red line) following the procedure of Statt (11). The solid red line with squares is the Morse gel line determined from rheological quench data. The dashed green and blue curves are sketches of the percolation line and glass line extending into the phase instability region, respectively. Star symbols indicate where snapshots in (b) and (c) were taken. (b) Particle snapshots taken near the critical point determined from the coexistence simulations to confirm its location. The vertical dashed red line indicates the transition from the homogeneous fluid to the phase instability region. (c) Particle snapshots taken near T_g^{sp} to confirm the location of the dense branch of the phase boundary determined from coexistence simulations. Yellow circles indicate the location of particle free voids. The vertical dashed blue line indicates the transition from the phase instability to the homogeneous attractive glass region.

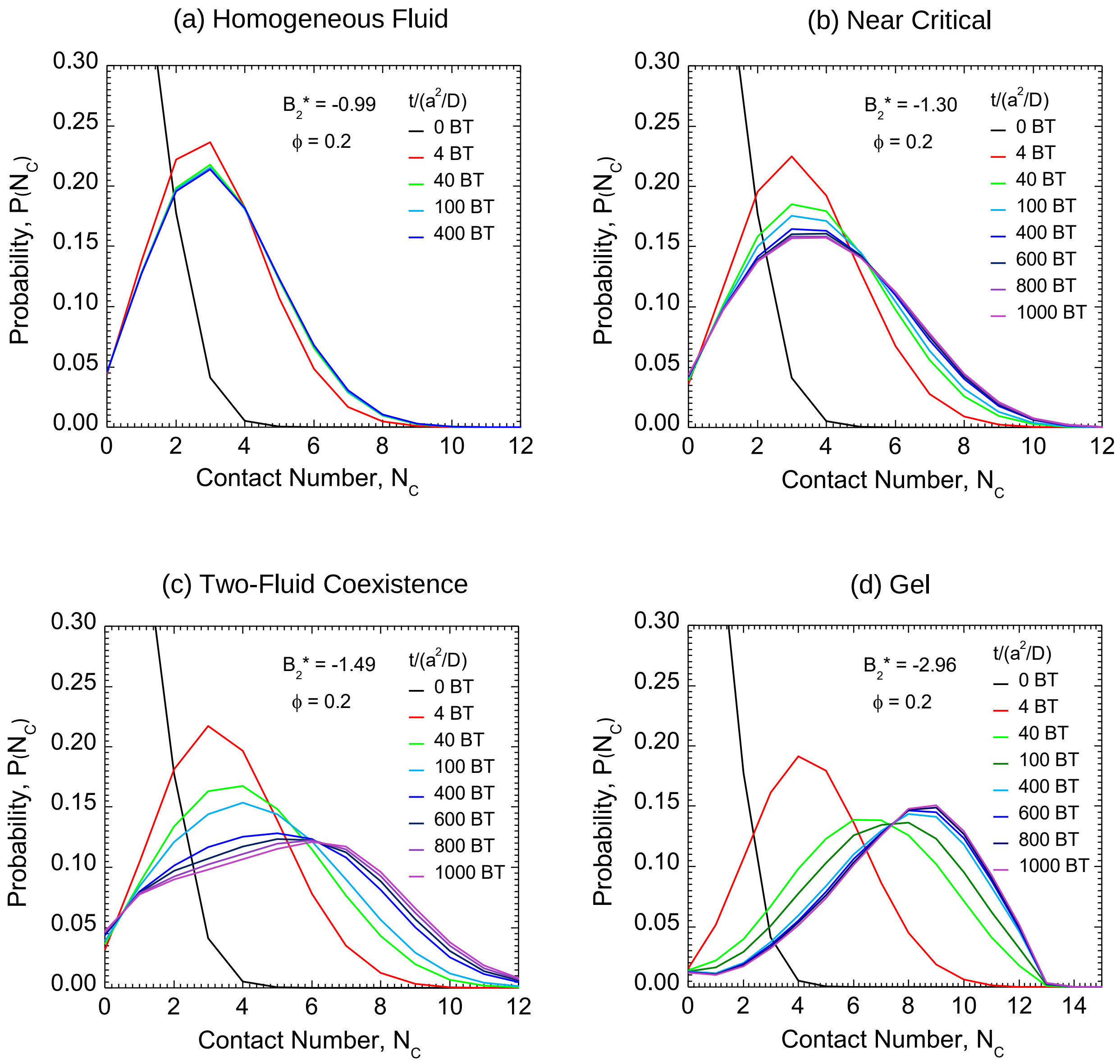

Fig. S11. Time evolution of particle contact number distributions for the Morse for 4 different quench depths at $\phi = 0.2$: (a) a quench in the homogeneous fluid phase (b) a quench to near the critical point (c) a quench to the two-fluid coexistence region between the phase boundary and the gelation line and (d) a quench below the gelation line.

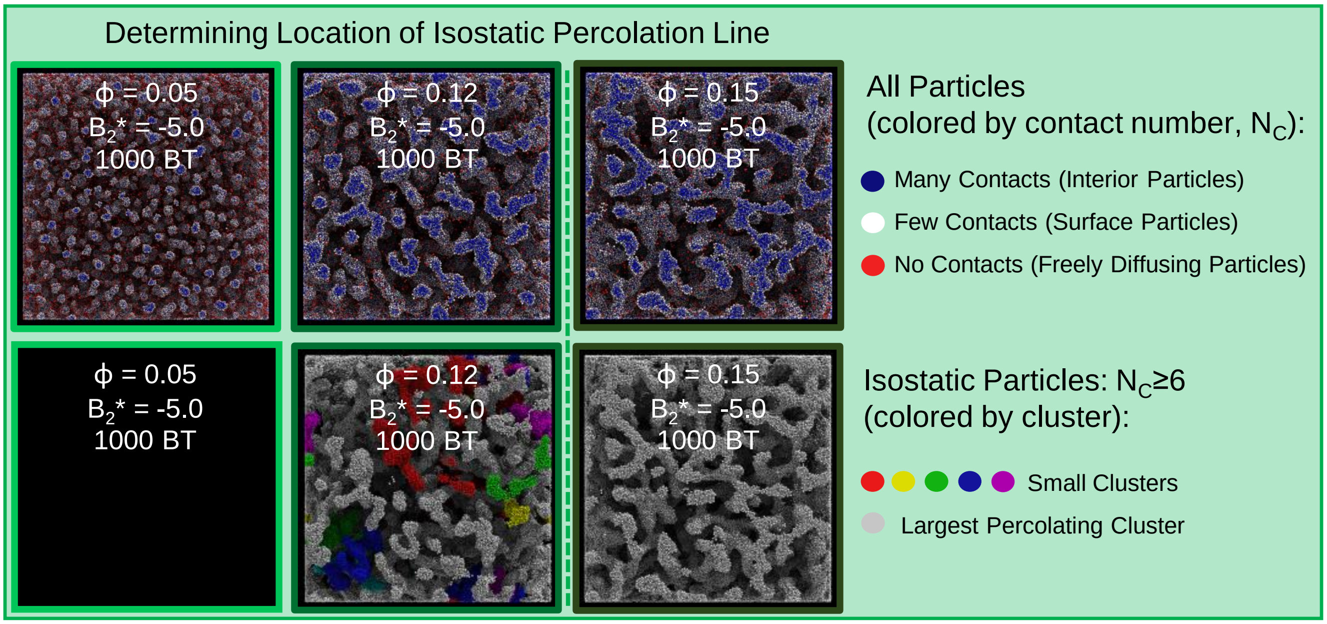

Fig. S12. Simulation snapshots at B_2^* = -5.0 and 1000 BT for ϕ = 0.05,0.12,0.15 taken to determine the location of the isostatic percolation line. The top row of snapshots show all particles colored according to contact number. The bottom row of snapshots show only isostatic particles ($N_C \geq 6$) after small clusters and non-isostatic particles ($N_C < 6$) have been removed. In the bottom row, distinct isostatic particle clusters are color coded for ease of visualization. The largest isostatic cluster is colored gray.

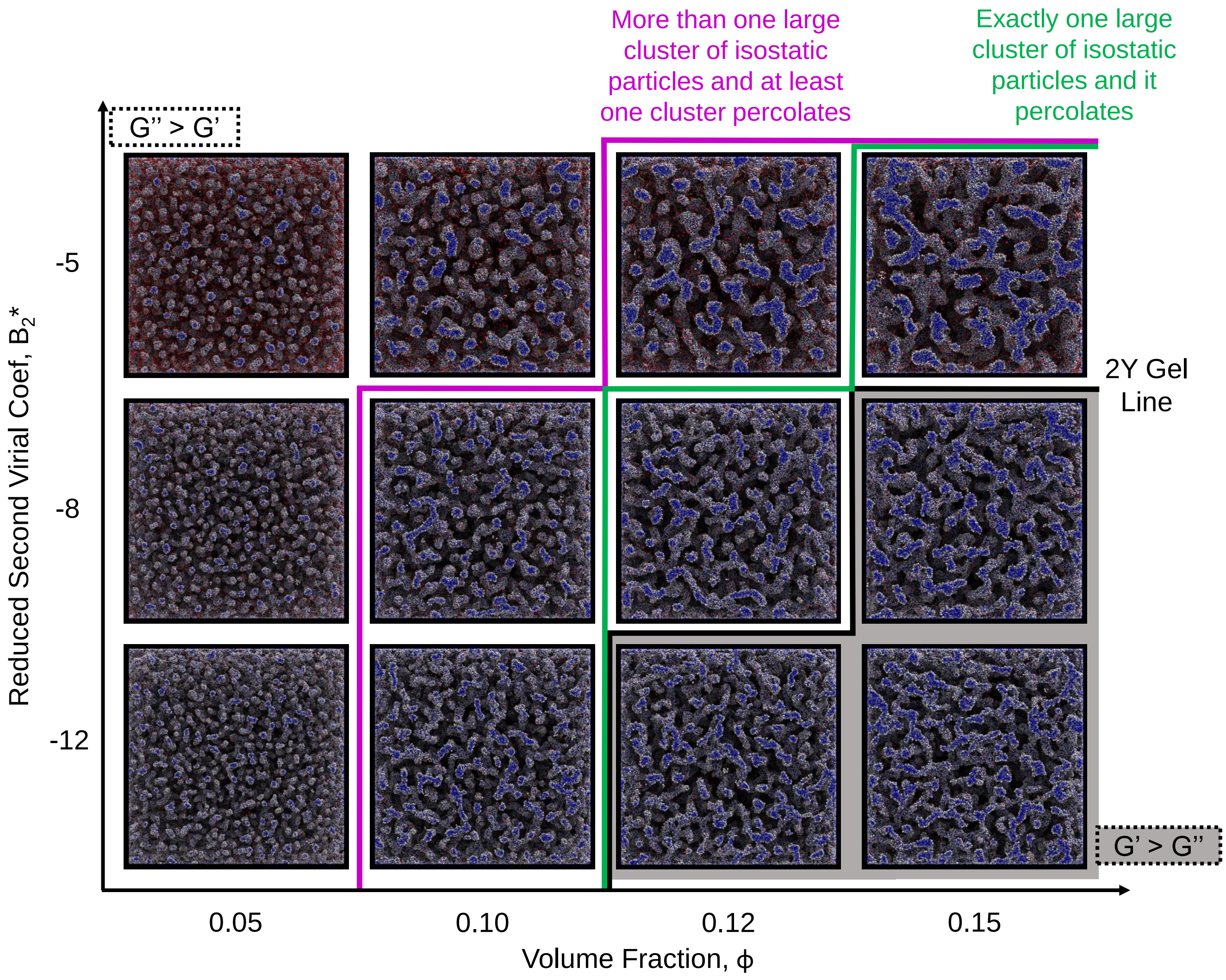

Fig. S13. All simulation snapshots taken at 1000 BT to determine the location of the isostatic percolation line. The solid black line is the 2Y gel line determined from rheological quench data. To the right of the black line $G' > G''$. The transition from discrete clusters to percolation of isostatic particles is demarcated by the pink line. As previously discussed, isostatic percolating networks can generally be classified into two categories: (1) where there exists multiple large clusters of isostatic particles and at least one of them percolates (this occurs to the right of the pink line and to the left of the green line) and (2) where there exists exactly one large cluster of percolating isostatic particles (this occurs to the right of the green line) . (2) is used to define the isostatic percolation threshold (green line) as it is a more stringent condition and corresponds more closely with the gelation transition (black line).

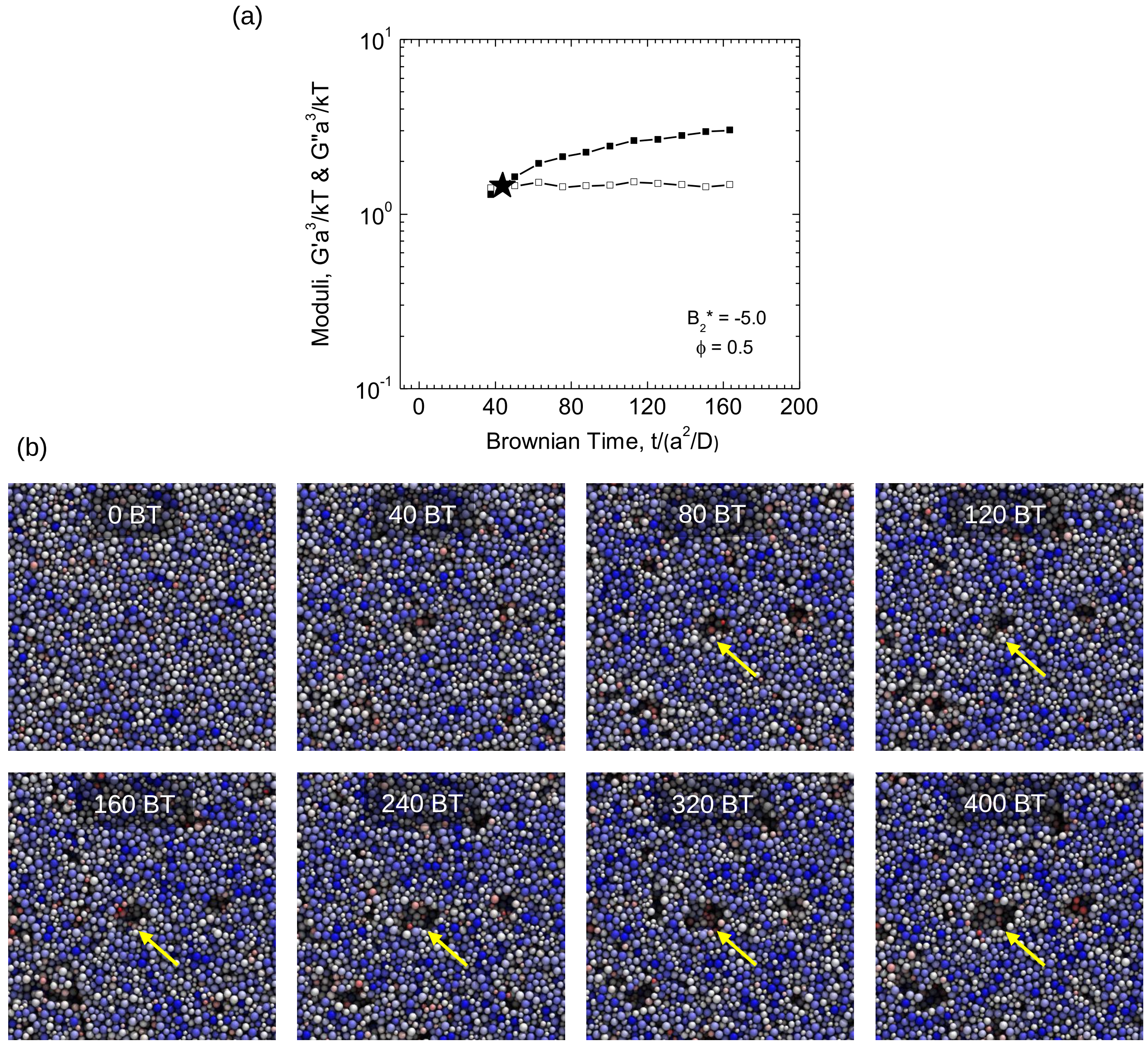

Fig. S14. Time evolution of 2Y viscoelastic moduli and simulation snapshots for an instantaneous quench into the bubbling glass region (region VII, $B_2^* = -5.0$ and $\phi = 0.5$) show the formation of a heterogeneous glass. (a) Time evolution of the linear viscoelastic moduli, G' (closed black squares) and G'' (open black squares) after the quench. For quenches into region VII, gelation occurs nearly instantaneously ($\tau_{gel} \sim 40BT$ or within the first few oscillation cycles after the quench) as indicated by the black star. (b) Simulation snapshots taken after the quench reveal the evolution of microscructure from an initially homogeneous glass to a heterogeneous glass with particle free voids or "bubbles" forming as the system ages and evolves towards equilibrium.

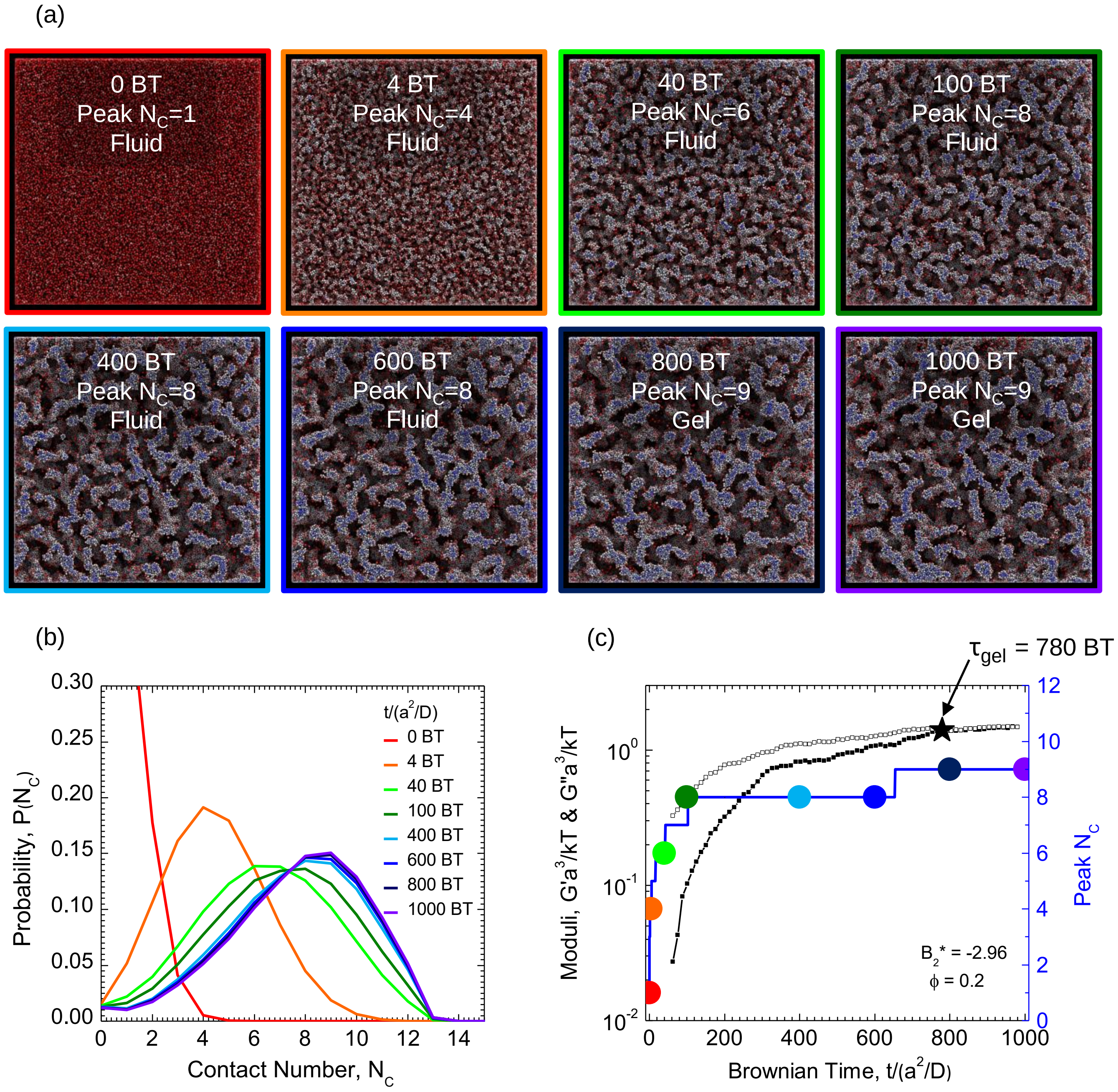

Fig. S15. Time evolution of Morse simulation snapshots, $P(N_C)$, and viscoelastic moduli for an instantaneous quench into the coarsening glassy gel region (region VI, $B_2^* = -2.96$ and $\phi = 0.2$) show gelation corresponding with the formation of an attractive glass. (a) Simulation snapshots taken before and after gelation ($\tau_{gel} \sim 780BT$). (b) Time evolution of $P(N_C)$. (c) Left axis: Time evolution of the linear viscoelastic moduli, G' (closed black squares) and G'' (open black squares) after the quench. The black star symbol indicates the gel time ($\tau_{gel} \sim 780BT$). Right axis: Time evolution of the N_C location of the peak in the $P(N_C)$ distributions from (b) (blue line). The colored circle symbols indicate the N_C location of the peak in $P(N_C)$ at times corresponding with $P(N_C)$ curves in (b) and simulation snapshots in (a). The emergence of elasticity (black star) coincides with the appearance of a peak at $N_C = 9$ indicating that the formation of an attractive glass in the particle dense strands dramatically slows further evolution of structure and arrests phase separation.

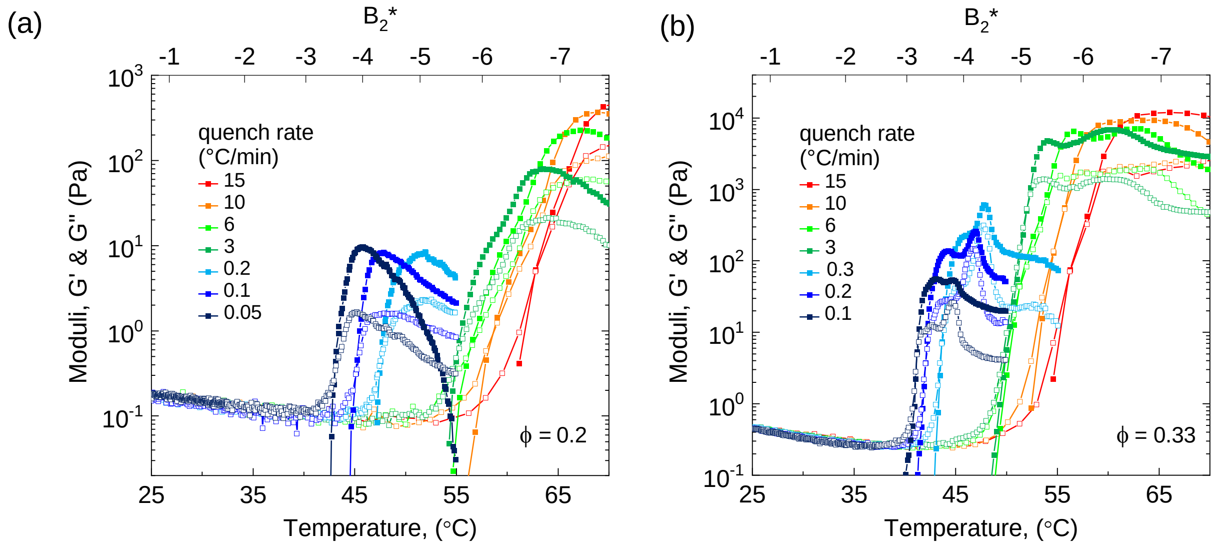

Fig. S16. Effect of ramp rate on G' (closed symbols) and G'' (open symbols) for the experimental system for two colloidal volume fraction, (a) $\phi = 0.2$ and (b) $\phi = 0.33$. Temperature ramps start at $T = 25^{\circ}C$ ($B_2^* = -0.80$, region II in the experimental state diagram) and ramp at different rates (in $^{\circ}C/min$) into the phase instability region (region V) before crossing the gelation line and entering region VI. A linear temperature ramp represents a nonlinear ramp in B_2^*, so a nonlinear B_2^* axis has been added to the top of each plot.

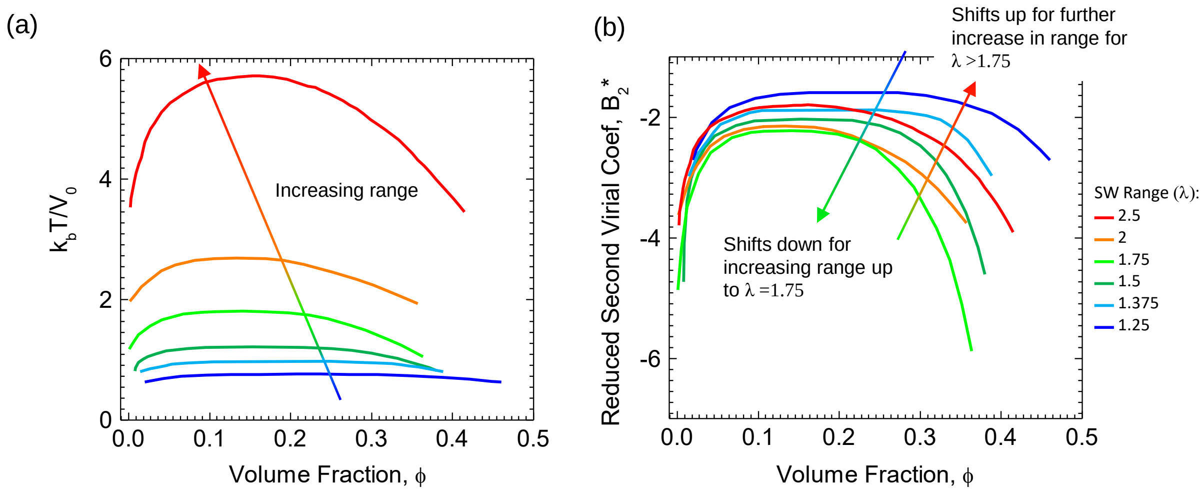

Fig. S17. Square well phase envelopes adapted from del Rio (5) and El Mendoub (21) for various SW ranges, λ, shown in two different representations: (a) k_bT/V_0 vs ϕ, and (b) B_2^* vs ϕ.

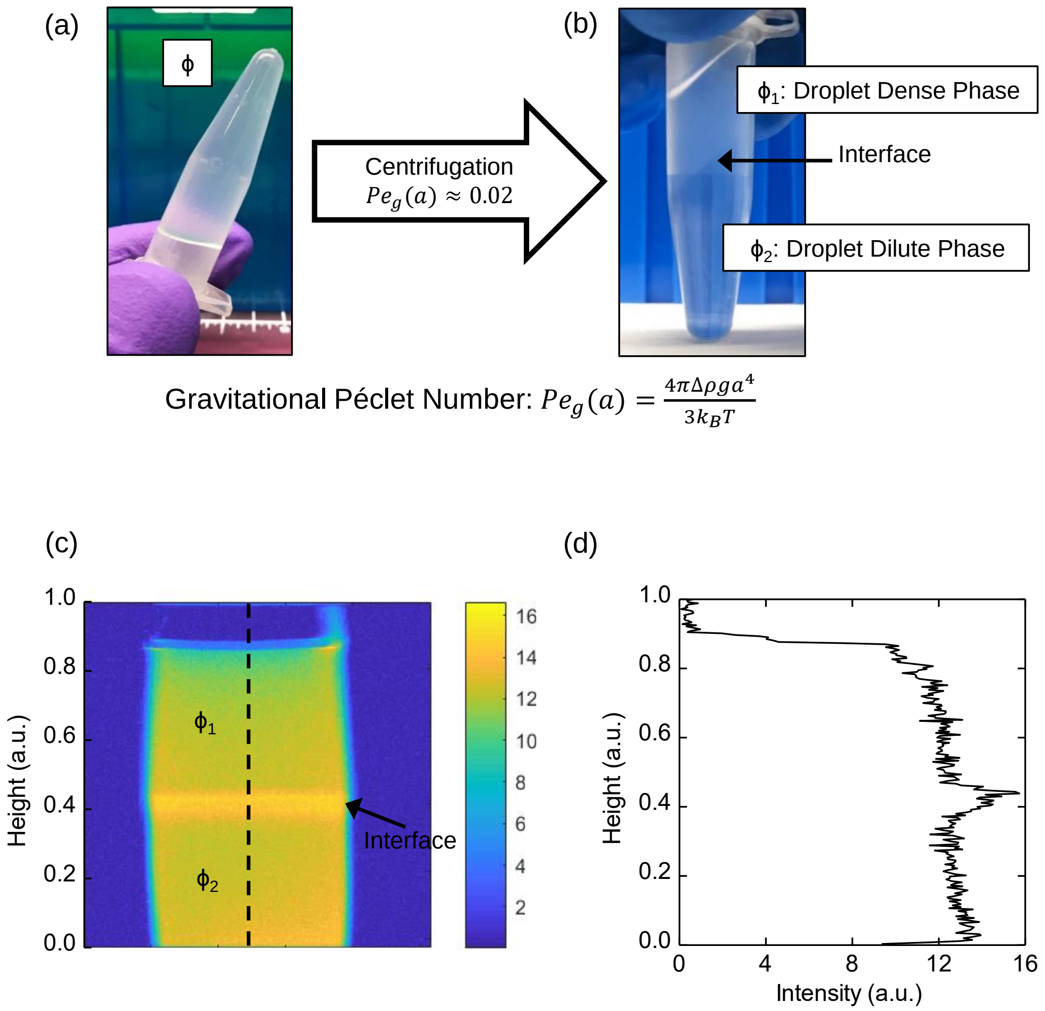

Fig. S18. Centrifugation of experimental colloidal gel to determine PEGDA distribution in dense and dilute phases. (a) image of a $\phi = 0.33$ colloidal gel formed in a centrifuge tube placed in an oven at $50^\circ C$ ($B_2^* = -4.75$) for 30 minutes. (b) The gel from (a) after being centrifuged at 9000 x g (gravitational Péclet number, $Pe_g(a) \approx 0.02$ where $\Delta\rho$ is the density difference between the dilute and dense phases, and g is the gravitational acceleration) for 20 minutes. Two distinct phases are visible, a cloudy droplet rich phase on top with droplet volume fraction ϕ_1, and a clear droplet dilute phase on bottom with droplet volume fraction ϕ_2. The interface between the two phases is indicated with an arrow. (c) Distribution of PEGDA in the droplet dense and droplet dilute regions determined with a magnetic resonance imaging (MRI) scan of a vertical slice of the centrifuge tube in (b) at the frequency of the ethylene glycol proton shift on the PEGDA. The interface between the droplet rich and droplet dilute phases is indicated with an arrow. (d) Intensity profile along the dashed line in (c) at the center of the centrifuge tube.

References

1. MJ Pagenkopp, TG Mason, Surfactant partitioning in nanoemulsions. *Langmuir* **34**, 10309–10320 (2018).
2. LC Hsiao, PS Doyle, Celebrating soft matter's 10th anniversary: Sequential phase transitions in thermoresponsive nanoemulsions. *Soft Matter* **11**, 8426–8431 (2015).
3. ME Helgeson, SE Moran, HZ An, PS Doyle, Mesoporous organohydrogels from thermogelling photocrosslinkable nanoemulsions. *Nat. materials* **11**, 344–352 (2012).
4. R Harich, et al., Gravitational collapse of depletion-induced colloidal gels. *Soft Matter* **12**, 4300–4308 (2016).
5. F Del Rio, et al., Vapour—liquid equilibrium of the square-well fluid of variable range via a hybrid simulation approach. *Mol. Phys.* **100**, 2531–2546 (2002).
6. L Vega, E de Miguel, LF Rull, G Jackson, IA McLure, Phase equilibria and critical behavior of square-well fluids of variable width by gibbs ensemble monte carlo simulation. *The J. chemical physics* **96**, 2296–2305 (1992).
7. JR Elliott, L Hu, Vapor-liquid equilibria of square-well spheres. *The J. chemical physics* **110**, 3043–3048 (1999).
8. S Netemeyer, ED Glandt, Percolation behavior of the square-well fluid. *The J. chemical physics* **85**, 6054–6059 (1986).
9. H Tsurusawa, M Leocmach, J Russo, H Tanaka, Direct link between mechanical stability in gels and percolation of isostatic particles. *Sci. advances* **5**, eaav6090 (2019).
10. JC Maxwell, L. on the calculation of the equilibrium and stiffness of frames. *The London, Edinburgh, Dublin Philos. Mag. J. Sci.* **27**, 294–299 (1864).
11. A Statt, H Casademunt, CP Brangwynne, AZ Panagiotopoulos, Model for disordered proteins with strongly sequence-dependent liquid phase behavior. *The J. chemical physics* **152**, 075101 (2020).
12. E Zaccarelli, WC Poon, Colloidal glasses and gels: The interplay of bonding and caging. *Proc. Natl. Acad. Sci.* **106**, 15203–15208 (2009).
13. J Bergenholtz, M Fuchs, Nonergodicity transitions in colloidal suspensions with attractive interactions. *Phys. Rev. E* **59**, 5706 (1999).
14. K Dawson, et al., Higher-order glass-transition singularities in colloidal systems with attractive interactions. *Phys. Rev. E* **63**, 011401 (2000).
15. E Zaccarelli, Colloidal gels: equilibrium and non-equilibrium routes. *J. Physics: Condens. Matter* **19**, 323101 (2007).
16. JM Olais-Govea, L López-Flores, JB Zepeda-López, M Medina-Noyola, Interference between the glass, gel, and gas-liquid transitions. *Sci. reports* **9**, 1–7 (2019).
17. HH Winter, F Chambon, Analysis of linear viscoelasticity of a crosslinking polymer at the gel point. *J. rheology* **30**, 367–382 (1986).
18. F Chambon, HH Winter, Linear viscoelasticity at the gel point of a crosslinking pdms with imbalanced stoichiometry. *J. Rheol.* **31**, 683–697 (1987).
19. FJ Wegner, Corrections to scaling laws. *Phys. Rev. B* **5**, 4529 (1972).
20. G Orkoulas, AZ Panagiotopoulos, Phase behavior of the restricted primitive model and square-well fluids from monte carlo simulations in the grand canonical ensemble. *The J. chemical physics* **110**, 1581–1590 (1999).
21. E El Mendoub, JF Wax, I Charpentier, N Jakse, Integral equation study of the square-well fluid for varying attraction range. *Mol. Phys.* **106**, 2667–2675 (2008).
